# Deubiquitinase USP19 modulates apoptotic calcium release and endoplasmic reticulum stress by deubiquitinating BAG6 in triple negative breast cancer

**DOI:** 10.1002/ctm2.1398

**Published:** 2023-09-12

**Authors:** Xiaoqiang Zhang, Xuyu Chen, Fangze Qian, Yanhui Zhu, Gao He, Junzhe Yang, Xian Wu, Hongfei Zhang, Xiafei Yu, Xiaoan Liu

**Affiliations:** ^1^ Breast Disease Center The First Affiliated Hospital of Nanjing Medical University Nanjing Jiangsu China; ^2^ Cancer Hospital of the University of Chinese Academy of Science (Zhejiang Cancer Hospital) Hangzhou China

**Keywords:** BAG6, Bcl‐2, endoplasmic reticulum stress, m^6^A, TNBC, USP19

## Abstract

**Background:**

Triple‐negative breast cancer (TNBC), a heterogeneous subtype of breast cancer (BC), had poor prognosis. Endoplasmic reticulum (ER) stress was responsible for cellular processes and played a crucial role in the cell function. ER stress is a complex and dynamic process that can induce abnormal apoptosis and death. However, the underlying mechanism of ER stress involved in TNBC is not well defined.

**Methods:**

We identified ubiquitin‐specific protease 19 (*USP19*) as a TNBC negative regulator for further investigation. The effects of USP19 on BC proliferation were assessed in vitro using proliferation test and cell‐cycle assays, while the effects in vivo were examined using a mouse tumorigenicity model. Through in vitro flow cytometric analyses and in vivo TUNEL assays, cell apoptosis was assessed. Proteomics was used to examine the proteins that interact with USP19.

**Results:**

Multiple in vitro and in vivo tests showed that USP19 decreases TNBC cell growth while increasing apoptosis. Then, we demonstrated that USP19 interacts with deubiquitinates and subsequently stabilises family molecular chaperone regulator 6 (*BAG6*). BAG6 can boost B‐cell lymphoma 2 (*BCL2*) ubiquitination and degradation, thereby raising ER calcium (Ca^2+^) levels and causing ER stress. We also found that the *N*_6_‐methyladenosine (m^6^A) “writer” methyltransferase‐like 14 (*METTL14*) increased global m^6^A modification.

**Conclusions:**

Our study reveals that USP19 elevates the intracellular Ca^2+^ concentration to alter ER stress via regulation of BAG6 and BCL2 stability and may be a viable therapeutic target for TNBC therapy.

## Graphical abstract

1. Based on multiple in vitro and in vivo tests, we found for the first time that USP19 decreased triple‐negative breast cancer (TNBC) cell growth while regulating apoptotic calcium release. These results linked USP19 to BC carcinogenesis with apoptotic calcium release and endoplasmic reticulum stress, provided new insights into the mechanism of USP19 inhibiting BC carcinogenesis.

2. In vitro experiments with corresponding human triple negative BC cells (MDAMB‐231 and BT‐549) were used to verify the findings, we found that USP19 interacts with, deubiquitinates, and subsequently stabilises family molecular chaperone regulator 6 (BAG6) and BAG6 can boost B‐cell lymphoma‐2 (Bcl‐2) ubiquitination and degradation, thereby raising ER calcium (Ca^2+^) levels and causing ER stress.

3. Although neoadjuvant chemotherapy therapies for TNBC significantly improves the prognosis of patients, some patients still cannot benefit from them. In our study, we found that USP19 can effectively inhibited the progression of TNBC. We also found that the *N*_6_‐methyladenosine (m^6^A) “writer” methyltransferase‐like 14 (METTL14) increased global m^6^A modification. It was the important mechanisms by which USP19 inhibited breast cancer carcinogenesis.

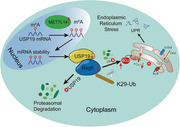

## 1 INTRODUCTION

Breast cancer (BC) is the most often diagnosed cancer in the world.^1^ TNBC, a heterogeneous subtype of BC, lacks expression of progesterone receptor, estrogen receptor, and human epidermal growth factor receptor 2.^2^, ^3^, ^4^ Despite the fact that TNBC accounts for 15%−20% of BC incidences, it was responsible for 25% of fatalities. Therapeutic interventions for TNBC are restricted, due to the absence of targetable receptors that differentiate other subtypes. Individuals with TNBC had poor prognosis and overall survival because of higher recurrence rate (over 30%) and shorter survival after metastatic recurrence.^5^ As a result, understanding the molecular and cellular processes involved in TNBC tumorigenesis is critical.

Ubiquitin‐specific protease 19 (*USP19*) is a ubiquitin‐specific protease localised in the endoplasmic reticulum (ER), which participates in the regulation of unfolded protein response (UPR).^6^, ^7^ Through interacting with HSP90 via the CS/p23 domains, *USP19* may promote HSP90 folding by giving it the ability to function as an independent partner.^8^ Jin et al.^9^ found that USP19 stabilises Beclin‐1 to promote autophagy, by removing the K11‐linked ubiquitin chain from Beclin‐1 at lysine 437, while reduces type I IFN signalling by blocking the interactions between RIG‐I‐MAVS. Cao‐Qi Lei al.^10^ found that C607S, an enzyme‐inactive mutant site of *USP19*, failed to inhibit *TNFA* and IL‐1β‐triggered *NF‐κB* activation after mutation. Even though there was evidence connecting *USP19* to the progression of BC, only a handful of the underlying molecular pathways have been discussed.^11^, ^12^, ^13^

ER is an important organelle responsible for protein processing, modification and folding, and plays a crucial role in cell Apoptosis.^14^ Different oncogenic factors, as well as metabolic and transcriptional abnormalities in the tumor microenvironment cooperate to disrupt ER homeostasis and immune cell infiltration, which trigger a persistent state of ER stress.^15^, ^16^, ^17^ It was generally believed that ER stress sensors activate three signalling pathways: Protein Kinase RNA‐like ER Kinase (PERK), Inositol‐Required Enzyme 1 (IRE1), and Activating Transcription Factor 6 (ATF6).^18^ Activated IRE1^19^ splices X‐Box‐Binding Protein 1 (*XBP1*) mRNA, which can create a powerful transcription factor. *XBP1* regulates the expression of multiple genes involved in quality control, protein folding and lipid synthesis in response to ER stress.^20^, ^21^ Logue^22^ reported that inhibition of IRE1 RNase, an ER stress sensor associated with ER stress resolution, can enhance paclitaxel‐mediated tumour suppression, and postpone tumour recurrence after treatment for TNBC. When it comes to ER stress, the UPR is an adaptive response to proteostatic stress (PR).^18^, ^23^ Shapiro^24^ showed that the anticipatory UPR pathway can be pre‐activated by peptide hormones, mitogenic steroids, and other effectors, which afterwards cause cytotoxicity in estrogen receptor‐positive BC. Here, it was shown that ER stress and UPR are inhibited in TNBC, and play an important role in the tumorigenicity and development of this subtype of BC.

This study revealed that *USP19* was expressed in TNBC tissues at low levels. We also found that *USP19* overexpression inhibits cell proliferation, induces apoptosis, and regulates cell‐cycle assay in TNBC cells. In terms of the underlying mechanism, we identified that U*SP19* functions as a deubiquitinase that stabilises *BAG6* (BAG family molecular chaperone regulator 6, a major histocompatibility complex (MHC)^25^ that facilitates tail‐anchored protein entrance into the ER). BAG6 could serve as a ubiquitinase that poromoted B‐cell lymphoma‐2 (*BCL2*) degrade. This study found a significant increase in m^6^A modification level in mRNA of TNBC tissues. Overexpression of *METTL14* significantly decreased the expression level and half‐life of *USP19* mRNA. The *METTL14/USP19/BAG6/BCL2* axis increases ER stress and apoptosis in TNBC cells through influencing ER calcium channels. Our research identifies a plausible mechanism for TNBC development and offers a promising method for the treatment.

## 2 MATERIALS AND METHODS

### 2.1 Tissue samples

RNA sequencing files and routine clinical data of patients diagnosed with BC were collected from The Cancer Genome Atlas (TCGA) data (https://portal.gdc.cancer.gov/) and the Genotype‐Tissue Expression (GTEx) database. Then, we assembled dataset containing 1 401 samples and RNA‐seq data (Table S2) of 196 patients (Table S3) underwent modified radical mastectomy at the Department of General Surgery, First Affiliated Hospital of Nanjing Medical University, China. And the other deubiquitinating enzyme (DUB) genes annotation files were derived from RNA‐seq data from experiments. Tissues were stored in liquid nitrogen for extraction of RNA and protein.

### 2.2 Cell lines

The human BC cell lines MDA‐MB‐231, MDA‐MB‐468, BT549, Hs578T, MCF7, SK‐BR‐3, T47D and ZR‐75‐1 as well as the normal human breast epithelial cell line MCF10A and HEK‐293T (human embryonic kidney 293T) were used in this study. Subtypes of the analysed cell lines: Basal‐like: MDA‐MB‐468, MDA‐MB‐231, BT‐549 and Hs578T. Luminal: T47D, MCF‐7. HER2^+^: SK‐BR‐3. normal mammary epithelial: MCF‐10A and MCF‐12A. Cells were cultured in dulbecco's modified eagle's medium (DMEM) with 10% fetal bovine serum (WISENT, Canada) and 1% penicillin/streptomycin (Gibco, USA) at 37° in a humidified atmosphere of 5% CO_2_.

### 2.3 Cell and p**lasmids** transfection

Constructs expressing Myc‐tagged *USP19*, Myc‐tagged *USP19* C607S, *USP19* siRNA, Flag‐tagged *BAG6*, *BAG6* siRNA and *METTL14* were generated using ORFs clones with the Myc or N‐terminal Flag sequence (HEBIO, China). Plasmids coding for HA‐tagged ubiquitin‐Lys6, ‐Lys11, ‐Lys27, ‐Lys29, ‐Lys33, ‐Lys48 and ‐Lys63 were obtained from Genebay Biotech. Lentiviral constructs for *USP19* and *BAG6* were generated by Corues Biotechnology. Scrambled lentiviral vectors were used as negative controls.

### 2.4 RNA extraction and quantitative qRT‐PCR assays

RNA Extraction Kit (Vazyme) was used to extract pure RNA. We used reverse transcription system (Toyobo) to synthesise complementary DNA. qRT‐PCR was performed on an Applied Biosystems 7900 Fast Real‐Time PCR System using SYBR Green PCR master mix. We normalised gene expression levels against internal control and displayed relative gene expression levels using the 2^−ΔΔ^ *^CT^* method. (Table S1)

### 2.5 Fluorescence microscopy

On a confocal dish, cells were cultivated and immediately observed. Cells were cultured, fixed with 4% paraformaldehyde, washed with PBS, then stained with certain antibodies for immunofluorescence microscopy analysis. A Leica DMI3000 B confocal microscope with an oil‐immersion objective set at 100 (NA 1.4) was used to evaluate colocalisation images.

### 2.6 Cell proliferation, colony formation and 5‐Ethynyl‐2′‐ deoxyuridine (**EdU)** incorporation assay

Cell proliferation ability was measured using EdU assay kit (RiboBio, China). Treated cells were seeded into 24‐well plates (2 × 10^4^ cells/well) overnight. Cells with EdU reagent were then incubated at 37°C for 2 h according to the manufacturer's instructions, and after fixation, membrane rupture and Hoechest33342 (400 μl) were added for immunostaining. The green and red fluorescence signals were all observed in the 500−530 and 553−618 nm spectral ranges, respectively. Proliferation was analysed by observing the average number of cells in the three regions of each sample using a Leica microscope.

### 2.7 Fluorescence detection and imaging

Cells were treated for 30 min at 37°C with 2 umol/L Fluo‐4/AM (Beyotime) for Ca^2+^ imaging. The green and red fluorescence signals were all observed in the 500–530 and 553–618 nm spectral ranges, respectively (Leica microscope).

### 2.8 Flow cytometric

Apoptosis and cell‐cycle were analysed through flow cytometric (FCM) methods. Transfected cells were washed twice with PBS, then trypsinised and centrifuged at 1 200 rpm for 5 min, fixed with 75% ethanol, and stored overnight at −20°C. Cells were incubated with PI and Annexin V‐FITC according to the instructions. All antibody‐ labelled cells were assayed using a FACSVerse 8 flow cytometer (BD Biosciences), and data were analysed by FlowJo software (Version 10.6.1).

Fluo‐4 AM (Beyotime) was used to assess intracellular Ca^2+^ levels. The treated cells were cultured with a solution of Fluo‐4 AM (2 umol/L) at 37°C for 30 min before being evaluated by flow cytometry. FlowJo software was used to examine the data.

### 2.9 In vitro deubiquitination assays

First, endogenous *BAG6* was immunoprecipitated using anti‐*BAG6* antibody, followed by immunoblotting using ubiquitin antibody to assess endogenous BAG6 ubiquitination. HA‐K11 and HA‐K29 and Flag‐*BAG6* were then co‐transfected into HEK‐293T cells. MG132 treated cells to increase levels of ubiquitinated BAG6. Subsequently, ubiquitinated BAG6 protein was immunoprecipitated using anti‐*BAG6*, anti‐Flag antibodies and protein G beads for enrichment. The magnetic beads were boiled mixed with 1× SDS loading buffer and analysed by Western immunoblot analysis.

### 2.10 Western blot assay

Using Radioimmunoprecipitation Assay (RIPA) Lysis Buffer (Beyotime) to lysis cells and BC tissue. Proteins were separated by SDS‐PAGE, transferred onto PVDF membranes (Millipore), blocked with 5% BLOT‐QuickBlocker (Beyotime), and incubated overnight in primary antibodies at 4°C. Incubating membranes with secondary antibody (1:20 000; Bio‐Rad) for 120 min at room temperature, then adding enhanced chemiluminescence reagent (Millipore). Using Image J software (National Institutes of Health) to semi‐quantify receptor density of protein bands (Table S1).

### 2.11 GST pull‐down assay

After being extracted from *E. coli* BL21, the pure GST‐*USP19* protein was incubated with either the GST‐*BAG6* or GST‐*BCL2* proteins. Protein G beads were used to cleanly separate the GST proteins. Following thorough washing of the beads, immunoblotting analysis was used to identify the bound *USP19*, *BAG6* and *BCL2*.

### 2.12 Animal experiments

At 4 weeks old, 60 female BALB/c nude mice were divided into four groups at random (*n* = 6 per group). The flanks of the naked mice received bilateral subcutaneous injections of the stably transfected cell lines MDA‐MB‐231 and BT‐549. Vernier calipers were used to measure the bidimensional tumours every 5 days, and after 4 weeks, the mice were euthanised. Using the equation volume = (width^2^ length)/2, the volume of the implanted tumor was estimated. The ethical approval to perform animal experiments is 2019SR512 (December 10, 2019).

### 2.13 Measurement of m^6^A modification

EpiQuik m^6^A RNA Methylation Quantification Kit was used to measure the amounts of m^6^A in total RNA (P‐9005; Epigentek Group Inc., Farmingdale, NY, USA). 200 ng of RNA were added to the assay wells together with the m^6^A standard, and then capture and detection antibody solutions were added. By measuring each well's absorbance at a wavelength of 450 nm (OD450), the m^6^A levels were then calculated colorimetrically and determined using the standard curve.

### 2.14 Immunoprecipitation

Separated MDA‐MB‐231 and HEK‐293T cells were lysed in protease inhibitor‐containing RIPA lysis buffer (Beyotime). We measured protein concentrations using a bicinchoninic acid (BCA) protein assay kit (Beyotime). Protein A/G‐agarose beads (Beyotime) were used to preclear cell lysates for 0.5 h. Next, the lysates were immunoprecipitated with the appropriate antibodies overnight at 4°C. The lysates underwent a second 3‐hour incubation with protein A/G‐agarose beads the next day. The RIPA lysis solution was used to wash the immunocomplexes five times, after which the bound proteins were boiled to release them and exposed to SDS‐PAGE for Western blot analysis.

### 2.15 Immunoprecipitation coupled with mass spectrometry (IP/MS)

Total proteins were extracted from HEK‐293T cells and immunoprecipitated as previously mentioned using the proper primary antibody and protein A/G‐agarose beads (Beyotime). Mass spectrometry was used to assess the extracted immunoprecipitates.

### 2.16 Immunohistochemical analysis

All samples and tumours that were implanted were fixed in 4% formalin before being paraffin embedded. Sections (thickness, 4 m) were treated with primary antibodies for the specific detection of *USP19*, *BAG6*, *CHOP*, *GRP78* or *Ki67* (proteintech, China) for an overnight incubation at 4°C after blocking endogenous peroxides and proteins. Sections were treated with secondary antibodies that were HRP‐polymer‐conjugated for 1 h at 37°C. Sections were then stained for 3 min in 3,3‐diaminobenzidine solution and hematoxylin was used as a counterstain on the nuclei. Sections of tumour were evaluated in a blinded way. Based on three randomly chosen fields for each section, the percentage of tumours that tested positive and the degree of cell staining were calculated. A representative dataset is displayed as mean ± SEM values. ns., not significant, *p < .05, **p < .01, ***p < .001.

### 2.17 Bioinformatic analyses

By using the Bioconductor packages “DESeq2,” “limma,” and “GSEA,” as well as the R packages “DESeq2” and “GSEA”, we were able to identify differentially expressed genes (DEGs). We used Gene Set Enrichment Analysis (GSEA) enrichment analysis to look at the distinct RNA modification patterns in biological processes. GSEA software revealed that tumour hallmarks were more prevalent in the high‐expression group compared to the low‐expression group (normalized enrichment score (NES) > 1, *p*‐value < .05, false discovery rate (FDR) < .25).

### 2.18 TUNEL assay

The In Situ CellDeath Detection Kit was used in a terminal deoxynucleotidyl transferase‐mediated dUTP nickend labelling (TUNEL) experiment (Servicebio, China). Dewaxing, rehydrating, incubation and other steps of paraffin slices were done in accordance with procedure. The sample was treated with three washes of PBS before being treated with a TUNEL reaction mixture containing TdT and dUTP (1:9) and incubated at 37°C for 2 h in a humid environment. The samples were examined under a fluorescence microscope after being counterstained with DAPI. The formula used to determine the cancer cells’ apoptotic index was as follows: apoptotic index = apoptotic cells/total cells 100%.

### 2.19 Study approval

All animal experiments are in accordance with the Public Health Service Policy on Humane Care of Laboratory Animals and approved by Institutional Animal Care and Use Committee (IACUC) of Gempharmatech Co., Ltd (AP#: 2107052).

### 2.20 Statistics analysis

At least three distinct biological replicates are included in the data, which are displayed as mean SD ± SD. The statistical analysis was performed using GraphPad Prism version 8 (GraphPad Software) and R software (version 4.0.1). Prior to doing any parametric studies, we checked the distribution's normality and the equality of the variance. For comparisons between two groups, the unpaired, two‐tailed Student's *t*‐test was used, and for comparisons involving more than two groups, the one‐ or two‐way ANOVA was followed by the Tukey's post hoc test (normality and equal variance passed). When normality and/or equal variance failed, the Wilcoxon rank‐sum test and the Kruskal–Wallis test were used to analyse nonparametric data. Statistical significance was defined as a *p*‐value less than 0.05. Kaplan–Meier survival curves were also employed to compare the overall survival in two subgroups.

## 3 RESULTS

### 3.1 *USP19* was down‐regulated in triple‐negative breast cancer tissues and cells

**FIGURE 1:**
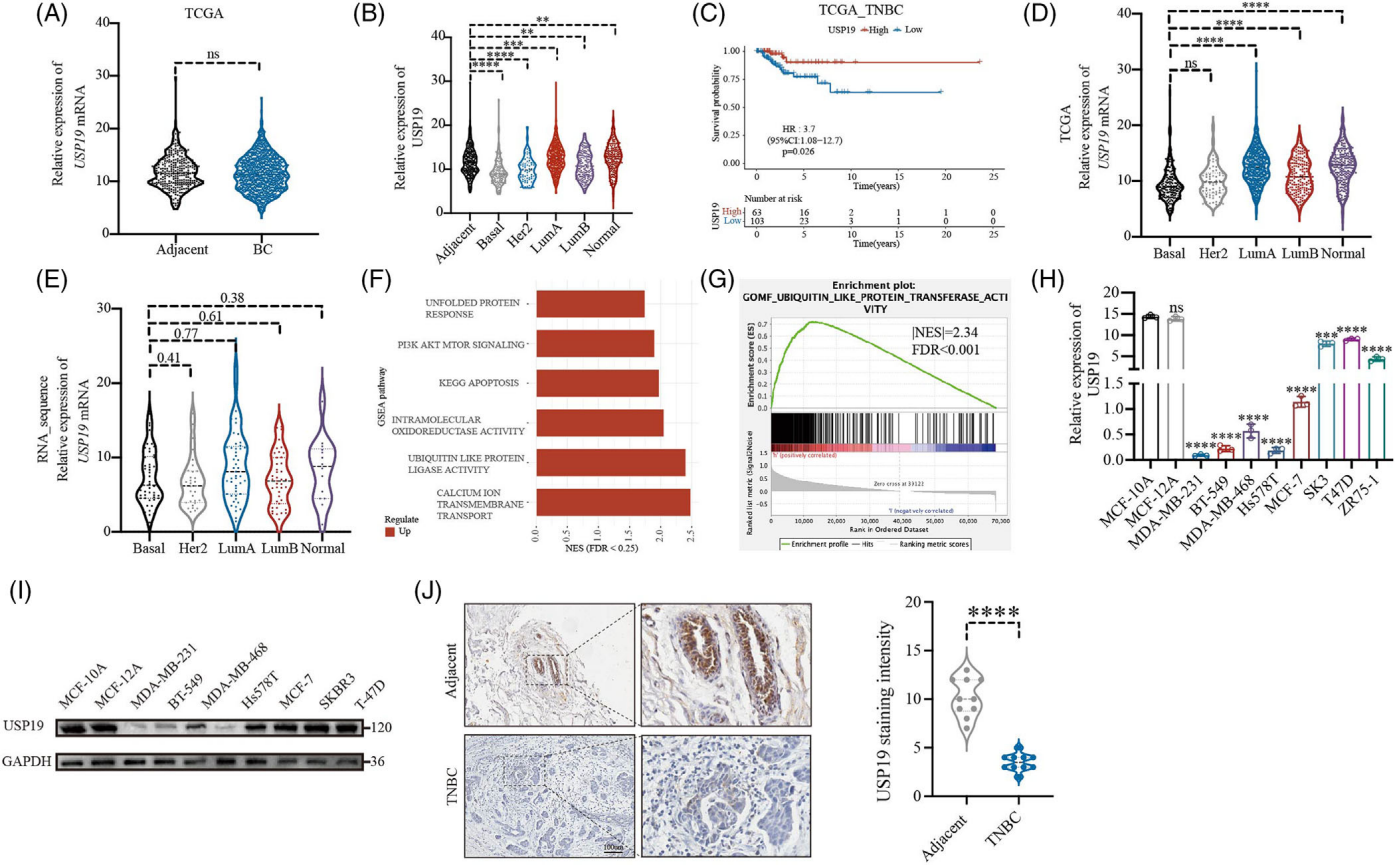
Expression of *USP19* in BC tissues, BC cells and transfected cells. (A,B) The expression of *USP19* in different tissues (Basal‐like, BC (breast cancer) and normal tissues) from TCGA (The Cancer Genome Atlas) and GTEx (Genotype‐Tissue Expression) cohort. (C) Kaplan–Meier curves showed the stratification analysis of the *USP19* in TNBC tissues. (D) The expression level of *USP19* in subtypes of BC in TCGA cohort. (E) The expression level of *USP19* in subtypes of BC in TCGA cohort and 196 breast cancer tissue samples RNA sequencing data. (F,G) Gene set enrichment analysis (GSEA) based on the sequencing data of 196 cases showed that calcium ion transmembrane transport, ubiquitin like protein ligase activity, intramolecular oxidoreductase activity, KEGG apoptosis, *PI3K AKT MTOR* signalling and unfolded protein response was enriched in the *USP19* high‐expression subgroup. (H,I) The expression levels of *USP19* in BC cells and MCF‐10A were detected by mRNA qRT‐PCR and Western blot. (J) Immunohistochemical staining against *USP19* collected from TNBC and adjacent tissue. A representative dataset is displayed as mean ± SEM values. ns., Not significant, *p < .05, **p < .01, ***p < .001.

To gain insight into the functions of ubiquitin modification in BC, we performed a screen interrogating expression data for 84 human DUB genes in TCGA^26^ and the Genotype‐Tissue Expression (GTEx) databases.^27^ After conducting an analysis of *USP19* expression in BC and adjacent tissues, our findings indicate that there was no significant difference in *USP19* expression between cancer and adjacent normal tissues (Figure 1A). Following that, we carried out an independent analysis to examine the expression of *USP19* across various subtypes of BC. Our findings revealed that the expression level of *USP19* was significantly lower in the basel‐like subtypes when compared to paracancer tissues (Figure 1B). Moreover, patients with high *USP19* expression exhibited a better prognosis (Figure 1C). The correlation result between the expression of USP19 and the prognosis of BC patients is provided in Figure S2C.We further examined *USP19* expression in different subtypes of BC in TCGA dataset. As shown in Figure 1D, *USP19* expression was lower in the basal‐like subtype than that in other subtypes of BC. This conclusion was confirmed in our centre, by sequencing 196 BC tissue samples (Figure 1E). According to GSEA, the group with high expression of *USP19* was more linked with calcium ion transmembrane transport, ubiquitin like protein ligase activity, intramolecular oxidoreductase activity, KEGG apoptosis, *PI3K AKT MTOR* signalling and UPR (Figure 1F,G). We further examined *USP19* expression in normal mammary epithelial cells (MCF‐10A) and BC cells lines (MDA‐MB‐231, MDA‐MB‐468, BT‐549, Hs‐578T, MCF‐7, SK‐BR‐3, T47D, and ZR75‐1) by qRT‐PCR and Western blot. *USP19* expression was lower in the TNBC cell lines than that is normal mammary epithelial cells (Figure 1H,I). In order to examine *USP19* expression in paraffin‐embedded TNBC tissues, an IHC assay was used. TNBC tissues had lower levels of USP19 protein expression when compared to adjacent tissues (Figure 1J). To examine its biological roles in TNBC, MDA‐MB‐231 and BT‐549 cells were selected for transfection with *USP19* siRNA and Myc‐tagged plasmid constructs. *USP19* was significantly regulated in MDA‐MB‐231 compared with the levels detected in the control groups (Figure S1F).

### 3.2 *USP*19 suppressed cell proliferation and induced apoptosis of BC cell lines

**FIGURE 2:**
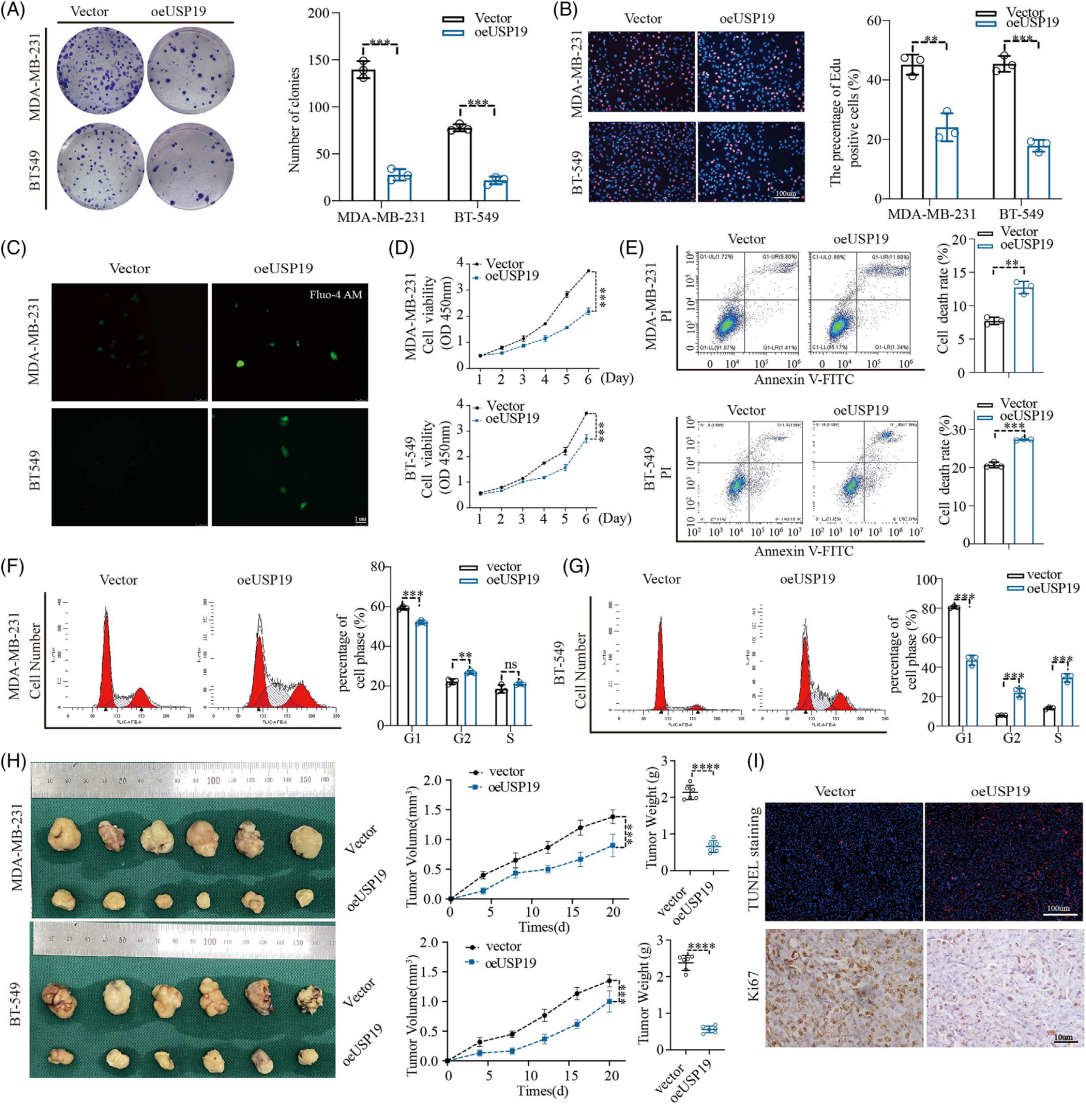
*USP19* inhibits cell proliferation and induces cell apoptosis. (A) Effects of *USP19* expression on the colony formation of BC cells. (B) Representative profiles of EdU cell growth in MDA‐MB‐231 cells and BT‐549 cells after transfection with Myc‐*USP19* plasmid respectively compared with the control. (C) Fluorescence microscopy was used to observe Fluo‐4 AM‐loaded MDA‐MB‐231 and BT549 cells. Fluo‐4 fluorescence (green) increases with intracellular Ca^2+^ concentration. (D) CCK‐8 was used to determine the proliferation of BC cells transfected with Myc‐*USP19* plasmid. OD value between Myc‐*USP19* plasmid and corresponding control group was significantly different. The data expressed as the mean ± SD. (E) Evaluation of the impact of altered *USP19* expression on cell apoptosis. (F,G) Effects of *USP19* modification on BC cell‐cycle distribution. (H) Photographs of tumours obtained from the different groups of nude mice transfected with Myc‐*USP19* plasmid, respectively. The curve graph exhibited the tumour growth measured at different time‐points after inoculation (*n* = 6). The image shows the tumour weight for each group (*n* = 6). (I) The TUNEL staining in tumor sections and IHC of *Ki67* in tumour sections (*n* = 3 for each group). Statistical results are quantified by ImageJ software. A representative dataset is displayed as mean ± SEM values. ns., Not significant, *p < .05, **p < .01, ***p < .001.

The colony formation assay was used to investigate the long‐term impact of *USP19* on cell proliferation. Results revealed that *USP19* overexpression impaired colony formation ability (Figure 2A). In addition, the 5‐ethynyl‐2′deoxyuridine (EdU) incorporation test was used to assess the effect of *USP19* on proliferation, in a more sensitive and specific manner. As shown in Figure 2B, the number of TNBC cells incorporating EdU in the *USP19*‐plasmid‐treated group was much lower than in the control group. Functionally, Fluo‐4 AM staining was used to assess intracellular Ca^2+^ levels. Fluorescence microscopy and flow cytometry revealed a substantial rise in intracellular Ca^2+^ concentration in *USP19*‐plasmid‐transfected cells, indicating that *USP19* could enhance Ca^2+^ levels (Figure 2C). The CCK‐8 method was then used to assess the effect of *USP19* on TNBC cell growth. In comparison to the control group, the proliferation rate of MDA‐MB‐231 and BT‐549 cells transfected with *USP19*‐plasmid was much lower (Figure 2D). The idea that *USP19* operates as a potential tumour suppressor gene in TNBC was confirmed by FCM measurement of apoptosis. When compared to the control group, cells overexpressing *USP19* had a greater apoptotic rate (Figure 2E). The results of the CCK‐8 cell viability assays matched those of the flow cytometry study. Based on these observations, we hypothesised that *USP19* promotes BC cell death. Flow cytometry was used to investigate the role of cell‐cycle disruption in the increased proliferation caused by *USP19* overexpression. We discovered that USP19 overexpression gradually reduced the proportion of cells in the G1 phase and increased the proportion of cells in the G2 and S phases, confirming the induction of cell cycle arrest in the G2 and S phases after USP19 overexpression (Figure 2F,G). BC cells that had been transduced with *USP19*‐targeting plasmid were injected into the flank of 5‐weeks old female nude mice to further demonstrate the tumour suppression role of *USP19* in vivo (Figure 2H). From the day 5 after injection, we began to calculate the tumor volume every 5 days until tumour sizes approached 1500 mm^3^ (day 20). Consistent with in vitro experiments, overexpression of endogenous *USP19* markedly attenuated both the tumour size and weight in vivo. The evidence that we stably transfected cell lines for in vivo experiments are provided in the new uploaded Figure S2A. TUNEL staining showed that the proportion of TUNEL‐positive cells increased in the oe*USP19* (overexpression *USP19*) group when compared with the control. Tumour samples from each group were stained for *Ki67*, demonstrating that *USP19* overexpression tumour‐bearing mice had much lower cell proliferation marker *Ki67* (Figure 2I). It was clear that tumour development rates in the *USP19* overexpression groups were much slower than those of the control groups, and tumour weights were also significantly reduced. These results unequivocally demonstrated the critical function of *USP19* in regulating TNBC cell proliferation. Subsequently, we investigated the impact of the C607S‐USP19 mutant and siUSP19‐2 on the proliferation and apoptosis of TNBC cells. Figure S1A–S1E presents the experimental findings.

### 3.3 *USP19* interacted with and stabilises *BAG6*

**FIGURE 3:**
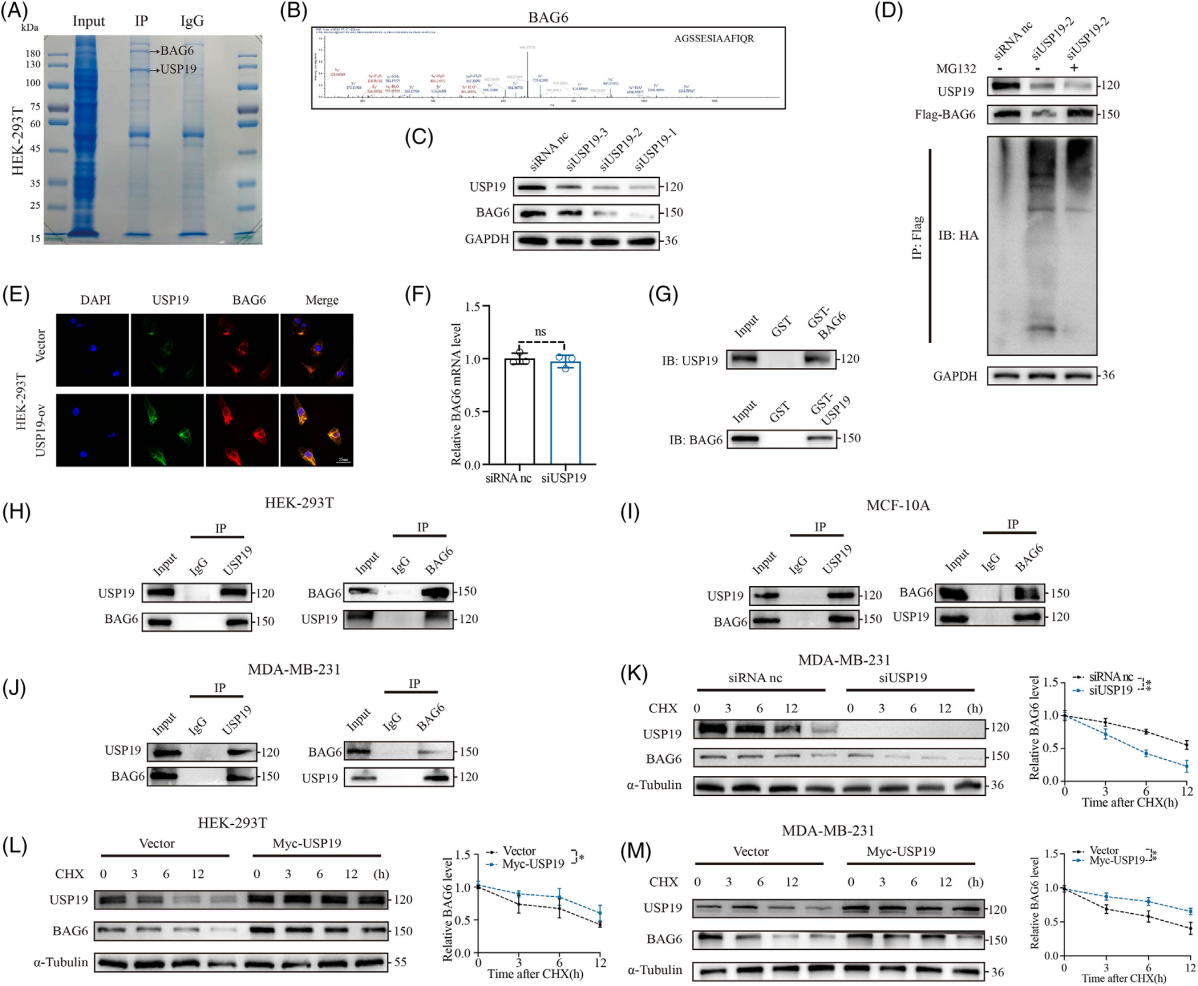
*USP19* interacts with and stabilises *BAG6*. (A) Coimmunoprecipitation and Coomassie brilliant blue staining analyses of *USP19* associated proteins in HEK‐293T cells. (B) IP/MS analysis indicated that *BAG6* is an interacting protein that binds to USP19. (C) Changes in endogenous BAG6 expression following si*USP19* transfection. (D) Analysis of BAG6, USP19 protein and ubiquitination levels by Western blot in HEK‐293T transfected with si*USP19*, either with or without proteasome inhibitor MG132. (E) HEK‐293T cells were incubated in the DMEM medium for 12 h after transfection Myc‐*USP19* plasmid for 48 h. Cells were imaged for Cy3‐Alexafluor‐488 co‐localisation. (F) *BAG6* mRNA level in HEK‐293T transfected with control siRNA and si*USP19*. (G) Endogenous protein interactions were confirmed in HEK‐293T lysates by immunoprecipitation with anti‐*USP19*, followed by immunoblotting with anti‐BAG6 or USP19, respectively. The interaction between USP19 and BAG6 was assessed using a GST pull‐down assay. All proteins were detected using the indicated antibodies. (H–J) The binding association of BAG6 and USP19 in cells by IP‐Western blot analysis in HEK‐293T, MCF‐10A and MDA‐MB‐231. (K–M) BAG6 protein levels in Vector and Myc‐*USP19* HEK‐293T and MDA‐MB‐231 were evaluated by immunoblotting with anti‐*BAG6* and anti‐*USP19* in the presence of cycloheximide (CHX, 10 μg/mL) for indicated timepoint. BAG6 protein levels in control siRNA and *USP19* siRNA MDA‐MB‐231 were evaluated by immunoblotting with anti‐*BAG6* and anti‐*USP19* in the presence of CHX (10 μg/mL) for indicated timepoint. A representative dataset is displayed as mean ± SEM values. ns., not significant, *p < .05, **p < .01, ***p < .001.

To identify putative interacting proteins for USP19, we constructed human embryonic kidney (HEK) 293T cells stably overexpressing *USP19*, by transfecting Myc‐tagged plasmids which would increase the interaction of proteins with USP19. We investigated which proteins interact with USP19 using immunoprecipitation/mass spectrometry (IP/MS), therefore elucidating the underlying mechanism of *USP19* in the regulation of TNBC cell proliferation and apoptosis. Numerous potential *USP19*‐interacting proteins were identified; among these proteins was BAG6 which combines with the tail‐anchored protein to recognise the hydrophobicity of the polypeptide synthesised by the ribosome, and promotes its entry into the ER. BAG6 simultaneously enhanced ubiquitin/proteasome‐mediated degradation of mis‐localised proteins, and was regarded as a putative interacting protein with USP19 (Figure 3A,B). We next researched the effect of knockdown of *USP19* on the *BAG6* expression levels in the MDA‐MB‐231 cell line. The endogenous BAG6 protein levels were downregulated in MDA‐MB‐231 cells after knocking down *USP19* (si*USP19*‐2 was selected for follow‐up experiments, Figure 3C). The reduction in BAG6 protein levels caused by *USP19* knockdown was reversed after treatment with proteasome inhibitor MG132. Western blotting was conducted using an anti‐ubiquitin antibody, demonstrating that the activity of MG132 functions as a proteasome inhibitor (Figure 3D). Knockdown of USP19 gene did not cause changes in BAG6 protein levels with lysosome inhibitor Chloroquine (CQ) (Figure S3B). We exclude the effect of USP19 on the lysosomal degradation pathway. Confocal imaging showed that the *BAG6* fluorescence signal was significantly enhanced after overexpression of *USP19*, which revealed the colocalization of BAG6 and USP19 (Figure 3E). Silencing and overexpressing *USP19* significantly reduced BAG6 protein levels, despite having no effect on *BAG6* mRNA levels (Figure 3F and Figure S3A), suggesting that *USP19* regulates *BAG6* not at the transcript but at the protein translation level. Additionally, the purified GST‐*USP19* and GST‐*BAG6* proteins produced in *E. coli* BL21 were used in an in vitro GST pull‐down test. BAG6 was subsequently treated with purified GST‐USP19 protein that had been immobilised on protein G beads (Figure 3G). Next, we examined the binding association of BAG6 and USP19 in cells by IP‐Western blot analysis. Ectopically expressed BAG6 was readily detected in the USP19 immune complex, and reverse coimmunoprecipitation (Co‐IP) confirmed that USP19 could also be detected in the BAG6 immune complex (Figure 3H). We also have conducted an IP/WB assay to showcase the interaction between USP19 and BAG6 in both breast normal and TNBC cell lines (MCF‐10A and MDA‐MB‐231) (Figure 3I,J). We also explored whether BAG6 interacts with USP19 mRNA directly. This hypothesis was negated by RNA pull‐down and immunoprecipitation assays (Figure S2B.). We transfected HEK‐293T cells with plasmids tagged with Myc and Flag epitopes, and Co‐IP experiments confirmed that Myc‐tagged *USP19* can co‐precipitate with Flag‐tagged *BAG6* (Figure S2D). We investigated the effect of *USP19* (up‐ or down‐regulation) on the stability of endogenous BAG6 protein level in the presence of the protein synthesis inhibitor cycloheximide (CHX). Overexpression of *USP19* significantly inhibited the degradation of BAG6, whereas knockdown of *USP19* markedly promoted BAG6 degradation (Figure 3K–3M and Figure S2E).

### 3.4 *USP19* removed the K29‐linked ubiquitin chains of BAG6

**FIGURE 4:**
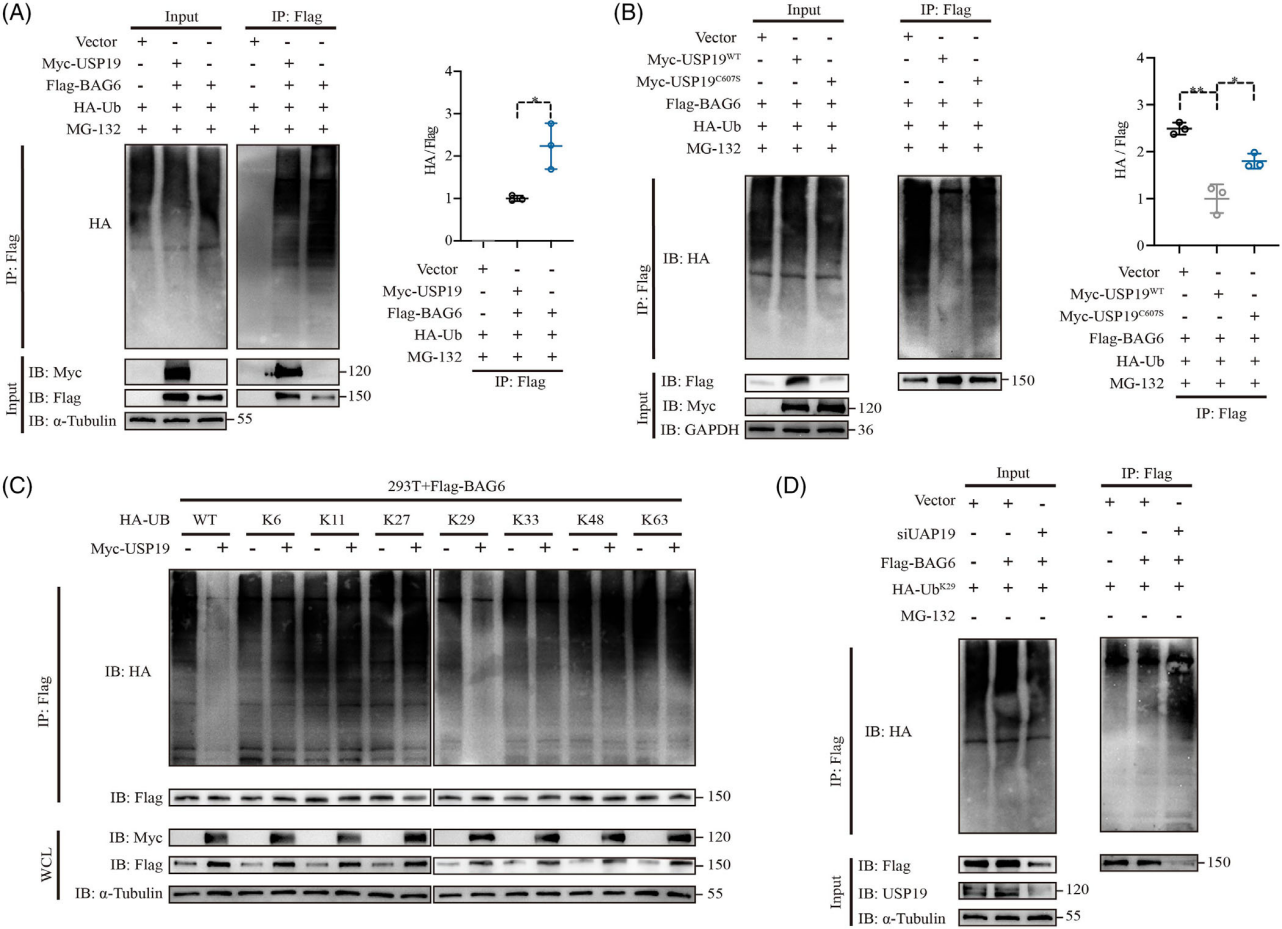
USP19 inhibits BAG6 ubiquitination. (A) HEK‐293T cells were transfected with Myc‐tagged *USP19*, Flag‐*BAG6*, HA‐UB or Vector and protein extracts were harvested after MG132 (10 μM) treatment for 3 h. Protein extracts were immunoprecipitated using anti‐flag antibody and analysed by immunoblot using anti‐HA and, anti‐Myc, anti‐flag and anti‐α‐Tubulin antibodies. (B) Lysates from HEK‐293T cells transfected with Myc‐tagged *USP19* (WT) or Myc‐tagged *USP19* (C607S), together with HA‐tagged Ub and Flag‐tagged *BAG6*, were immunoprecipitated with anti‐flag and immunoblotted with anti‐HA, anti‐Myc and anti‐flag. (C) Lysates of HEK‐293T cells transfected with plasmid expressing Flag‐*BAG6* and HA‐tagged ubiquitin (HA‐Ub (wild type), K6‐linked‐Ub, K11‐linked‐Ub, K27‐linked‐Ub, K29‐ linked‐Ub, K33‐linked‐Ub, K48‐linked‐Ub, or K63‐linked‐Ub), together with the empty vector or expression vector of Myc‐*USP19* and treated with MG132 (10 μM) for 3 h, were immunoprecipitated with anti‐flag and immunoblotted with anti‐HA. D. si*USP19*, a plasmid expressing flag‐*BAG6*, and K29‐linked‐Ub were transfected into HEK‐293T cells. Protein was extracted after being exposed to MG132 (10 μM) for 3 h, and it was then immunoprecipitated with anti‐flag and immunoblotted with anti‐HA. A representative dataset is displayed as mean ± SEM values. ns., not significant, *p < .05, **p < .01, ***p < .001.

We also investigated whether USP19 acts as a deubiquitination enzyme to stabilise BAG6 expression. We used immunoblot analysis with an anti‐Flag antibody to analyse the function of USP19 as deubiquitinase of BAG6, followed by immunoprecipitation with anti‐HA for total cell ubiquitin. We found that USP19 regulates the BAG6 level through deubiquitination modification. Overexpression of *USP19* significantly decreased ubiquitination of BAG6, but increased BAG6 protein level when compared with the control group (Figure 4A). To further clarify the regulatory role of BAG6 ubiquitination by USP19, MDA‐MB‐231 cells were co‐transfected with Myc‐*USP19* (wild‐type, WT) or Myc‐C607S mutant,^10^ Flag‐*BAG6*, and HA‐Ub. Next, to investigate whether the ability of USP19 to deubiquitinate BAG6 was dependent on its protease activity, we transfected WT‐*USP19* or its mutants into cells. Overexpression of WT‐*USP19* reduced BAG6 ubiquitination, but transfection with the C607S‐*USP19* plasmid had no effect on BAG6 ubiquitination (Figure 4B). Then, we investigated which types of BAG6 ubiquitination are regulated by USP19. Different processes of polyubiquitination, including ubiquitination of lysine 6 (K6)‐, K11‐, K27‐, K29‐, K33‐, K48 and K63‐linked diubiquitin, have been implicated in the regulation of intracellular protein levels.^28^, ^29^ Overexpression of *USP19* significantly inhibited the WT and K29‐linked polyubiquitination of BAG6, but did not affect the other ubiquitination linkages (K6, K11, K27, K33, K48 or K63) in BAG6 (Figure 4C). This was in agreement with *USP19* knockdown increasing K29‐linked ubiquitination of BAG6 (Figure 4D). In summary, these results demonstrated that the K29 linkage was essential for USP19 to exert its deubiquitination function and modify BAG6.

### 3.5 *BAG6* inhibition suppressed *USP19* overexpression‐induced events

**FIGURE 5:**
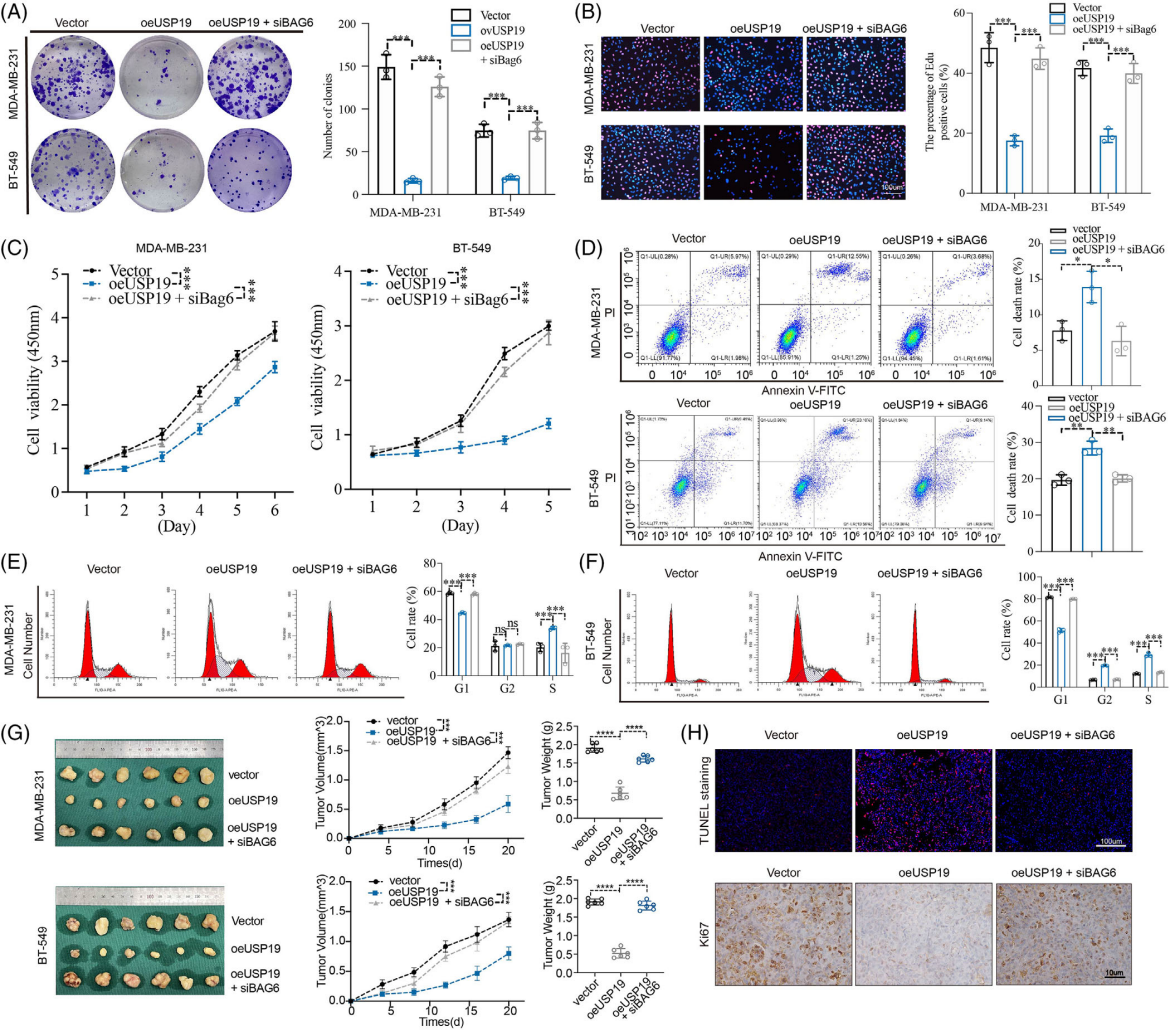
The roles of *USP19* and *BAG6* in the regulation of breast cancer cell proliferation and apoptosis. (A,B) *USP19* up‐regulation inhibits proliferation and induces apoptosis using the colony formation assay and EdU assay. The rescue experiments for *BAG6* knockdown were performed by *USP19* overexpression. (C) Similar rescue experiments for *BAG6* silencing were performed in CCK‐8 cell viability assays. (D–F) Cell viability assay, flow cytometry and the cell‐cycle test are used to demonstrate that *USP19* up‐regulation triggers apoptosis and reduces proliferation. By overexpressing *USP19*, *BAG6* knockdown rescue studies were carried out. (G) Photographs of mice tumours of each group (*n* = 6) at the end of the experiment. The curve graph exhibited the tumour growth measured at different time‐points after inoculation (*n* = 6). The image shows the tumour weight for each group (*n* = 6). (H) The TUNEL staining in tumour sections and IHC of *Ki67* in tumour sections (*n* = 3 for each group). A representative dataset is displayed as mean ± SEM values. ns., not significant, *p < .05, **p < .01, ***p < .001.

We demonstrated that expression of *USP19* inhibited proliferation and induced apoptosis in TNBC cells, as well as promoting BAG6 protein expression by deubiquitinating modification. To further verify whether the effects of *USP19* on proliferation in TNBC cells were mediated by regulation of *BAG6*, we knockdowned *BAG6* in MDA‐MB‐231 and BT‐549 cells (Figure 5A–C). Besides, we supplemented the experiments on the effect of BAG6 overexpression on proliferation in cells and tumour growth in Figure 3C,D. Subsequently, we investigated the ability of BAG6 to counteract the effects of *USP19* upregulation. BC cells were co‐transfected with *USP19* plasmid and *BAG6* siRNA. As shown in flow cytometry (Figure 5D–F), knockdown of endogenous BAG6 inhibited cell apoptosis, while attenuating cell cycle arrest in the G2 and S phases, which was opposite of the effects of *USP19* overexpression. The results showed that *BAG6* knockdown effectively reversed the promotion of proliferation and suppression of apoptosis induced by *USP19* overexpression (Figure 5A–F). The above results confirmed our hypothesis, namely that *USP19* directly targets *BAG6* to inhibit BC cell proliferation and induce apoptosis.

To clarify the role of *USP19* in cancer cell suppression through regulation of *BAG6* in vivo, MDA‐MB‐231 cells with expressed oe*USP19*, oe*USP19*/si*BAG6*, and control plasmids (Figure 5F), were subcutaneously injected into the right armpits of BALB/c nude mice (6 mice per group). The evidence that we stably transfected cell lines for in vivo experiments are provided in the new uploaded Figure S2A. From the day 5 after injection, we began to calculate the tumour volume every 5 days until tumour sizes approached 1500 mm^3^ (day 20). Consistent with in vitro experiments, overexpression of endogenous *BAG6* markedly attenuated both the tumour size and weight in vivo. TUNEL staining showed that the proportion of positive cells increased in the oe*USP19* group when compared with the control (Figure 5H). Tumour samples from each group were stained for *Ki67*, demonstrating that tumour‐bearing mice had much more up‐regulation of the si*BAG6*‐induced cell proliferation marker *Ki67* (Figure 5H). From rescue experiments, we also found that the effect of *USP19* could be compensated with *BAG6* expression.

### 3.6 BAG6 targeted BCL2 proteins to increase intracellular calcium

**FIGURE 6:**
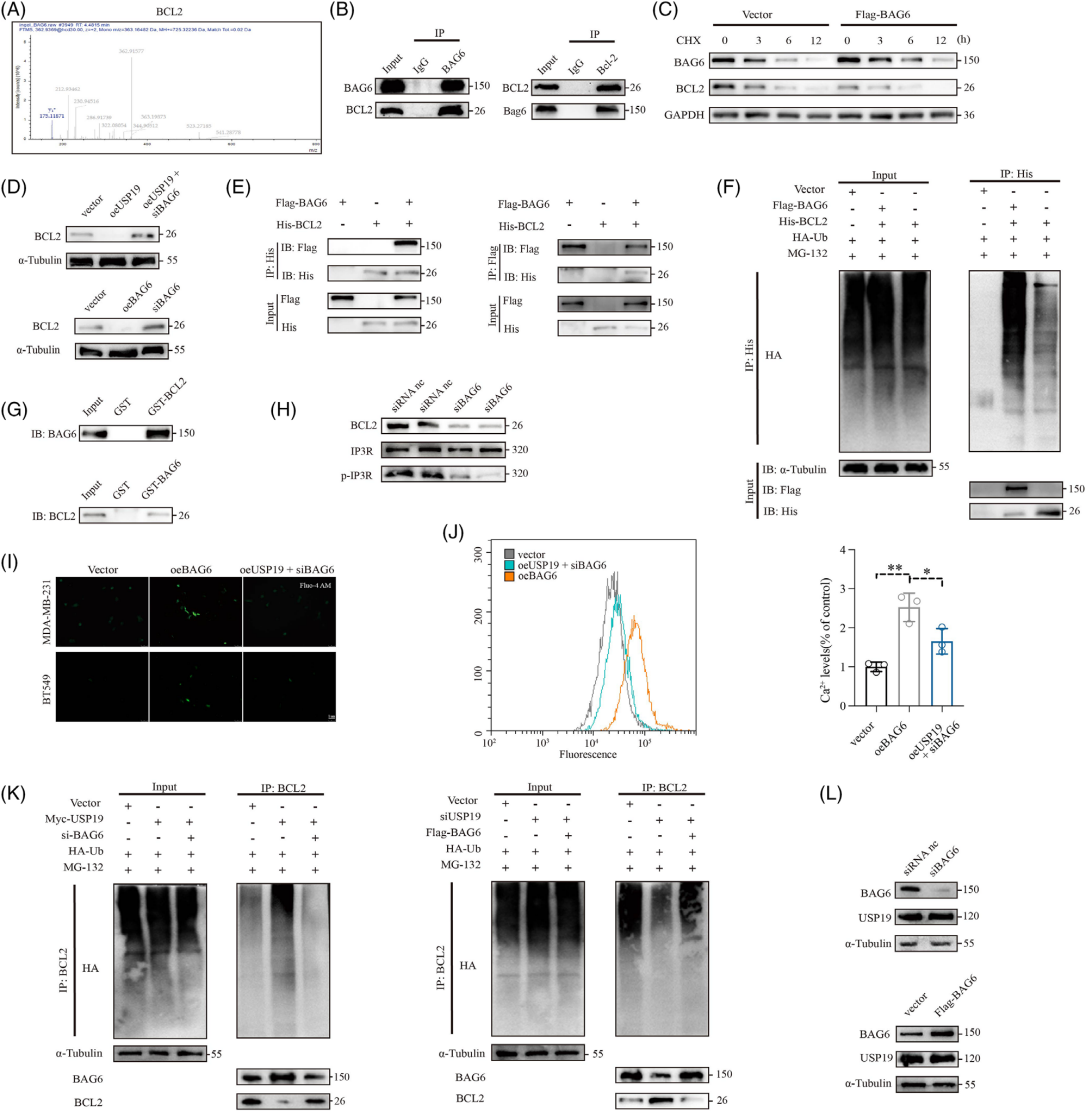
BAG6 interacts with and degrades BCL2. (A) IP/MS analysis indicated that BCL2 is an interacting protein that binds to BAG6. (B) Endogenous protein interactions were confirmed in MDA‐MB‐231 lysates by immunoprecipitation with anti‐*BAG6*, followed by immunoblotting with anti‐*BCL2* or *BAG6*, respectively. (C) BCL2 protein levels in Vector and Flag‐*BAG6* MDA‐MB‐231 were evaluated by immunoblotting with anti‐*BCL2* and anti‐*BAG6* in the presence of cycloheximide (CHX, 10 μg/mL) for indicated timepoint. (D) The impact of *USP19* and *BAG6* on BCL2 expression. (E)) Exogenous protein interactions were confirmed in MDA‐MB‐231 lysates by immunoprecipitation with anti‐*BAG6*, followed by immunoblotting with anti‐*BCL2* or *BAG6*, respectively. (F) MDA‐MB‐231 cells were transfected with Flag‐tagged *BAG6*, His‐*BCL2*, HA‐UB or Vector and protein extracts were harvested after MG132 (10 μM) treatment for 3 h. Protein extracts were immunoprecipitated using anti‐His antibody and analysed by immunoblot using anti‐HA and, anti‐Flag, anti‐His and anti‐α‐Tubulin antibodies. (G) The interaction between *BAG6* and *BCL2* was assessed using a GST pull‐down assay. All proteins were detected using the indicated antibodies. (H) Changes in endogenous IP3R and p‐IP3R expression following si*BCL2* transfection. (I) Fluorescence microscopy was used to observe Fluo‐4 AM‐loaded MDA‐MB‐231 and BT549 cells. Fluo‐4 fluorescence (green) increases with intracellular Ca^2+^ concentration. (J) Intracellular Ca^2+^ levels in the various groups were revealed by flow cytometry analysis. (K) MDA‐MB‐231 cells were transfected with Myc‐tagged *USP19*, si*USP19*, Flag‐*BAG6*, si*BAG6*, HA‐UB or Vector and protein extracts were harvested after MG132 (10 μM) treatment for 3 h. Protein extracts were immunoprecipitated using anti‐*BCL2* antibody and analysed by immunoblot using anti‐HA and, anti‐*BAG6*, anti‐*BCL2* and anti‐α‐Tubulin antibodies. (L) The impact of *BAG6* on USP19 expression. A representative dataset is displayed as mean ± SEM values. ns., not significant, *p < .05, **p < .01, ***p < .001.

Next, we further searched for the specific mechanism by which *BAG6* regulates TNBC progression. By transfecting flag‐tagged plasmids, which would enhance the interaction of proteins with BAG6, we created HEK‐293T cells that could consistently overexpress *BAG6*. By utilising IP‐MS, we looked at the proteins that interact with BAG6 in order to clarify the underlying mechanism of *BAG6* controls the course of TNBC (Figure 6A). His‐*BCL2* interacted with Flag‐*BAG6* according to Co‐IP and immunoblot analyses. The binding relationship between BCL2 and BAG6 in cells was then investigated using an IP‐Western blot analysis. The BAG6 immune complex was easily able to identify ectopically produced BCL2, and reverse Co‐IP indicated that BAG6 may also be found in the BCL2 immune complex (Figure 6B). The findings showed that *BAG6* overexpression significantly shortened the Bcl‐2 protein's half‐life in MDA‐MB‐231 cells (Figure 6C). The impact of *USP19* and *BAG6* on *BCL2* expression was then investigated on our further research. After overexpressing *USP19* and *BAG6*, the endogenous *BCL2* protein levels were reduced in MDA‐MB‐231 cells (Figure 6D). In MDA‐MB‐231 cells, Co‐IP with epitope‐tagged BAG6 and BCL2 was also carried out. Co‐transfection with Flag‐*BAG6* and His‐*BCL2* expressing plasmids was carried out in MDA‐MB‐231 cells to further demonstrate the link between BAG6 and BCL2. Results indicated that exogenous BCL2 might interact with ectopic BAG6 expression (Figure 6E). BAG6 is identified as a ubiquitin‐like protein^30^ that shuttles between the cytoplasm and nucleus. It was also involved in the regulation of apoptosis, antigen presentation and T‐cell response.^30^, ^31^ Furthermore, BAG6 could suppress RNA virus‐mediated innate immunity by increasing *VISA/TRAF2* K48 poly‐ubiquitination.^32^ Clearly, the upregulation of BAG6 greatly promoted BCL2 ubiquitination in MDA‐MB‐231 cells (Figure 6F). A GST pull‐down assay in vitro was performed using the purified GST‐BAG6 and GST‐BCL2 proteins generated in *E. coli* BL21. Following that, *BCL2* was treated with pure GST‐BAG6 protein that had been immobilised on protein G beads (Figure 6G). It has been observed that *BCL2* can control the activity of the intracellular Ca^2+^ channel inositol 1,4,5‐trisphosphate receptor (*IP3R*) and the release of calcium during apoptosis.^33^ ER stress, which compromises appropriate protein folding and intracellular transport, may be brought on by calcium homeostasis. The impact of *BCL2* knockdown on *IP3R* and *p‐IP3R* expression levels in the MDA‐MB‐231 cell line was the subject of our subsequent investigation. After *BCL2* knocking down, the endogenous p‐IP3R protein levels in MDA‐MB‐231 cells were reduced. But there was no substantial change in the *IP3R* expression (Figure 6H). Fluorescence microscopy and flow cytometry revealed a substantial rise in intracellular Ca^2+^ concentration in *USP19* plasmid and si*BAG6* transfected cells, indicating that *USP19/BAG6* axis could enhance Ca^2+^ levels through inhibiting *BCL2* expression (Figure 6I,J). Fluorescence microscopy revealed a substantial rise in intracellular Ca^2+^ concentration in *BCL2* plasmid transfected cells, indicating that *BCL2* could enhance Ca^2+^ levels in Figure S3E.

To further clarify the regulatory role of BCL2 ubiquitination by USP19 and BAG6, MDA‐MB‐231 cells were co‐transfected with Myc‐USP19, siUSP19, Flag‐BAG6, siBAG6 and HA‐Ub. According to the aforementioned findings, USP19 stabilises BAG6 protein, which in turn impacts ubiquitination of BCL2, but it has no effect on BCL2 protein's intrinsic ubiquitin level (Figure 6K). The expression level of USP19 remained unchanged after overexpressing or knocking down the BAG6 protein, indicating that BAG6 had no impact on the protein level of USP19 (Figure 6L).

### 3.7 *USP19* promoted apoptosis through *BAG6*‐induced ER stress in human BC cells

**FIGURE 7:**
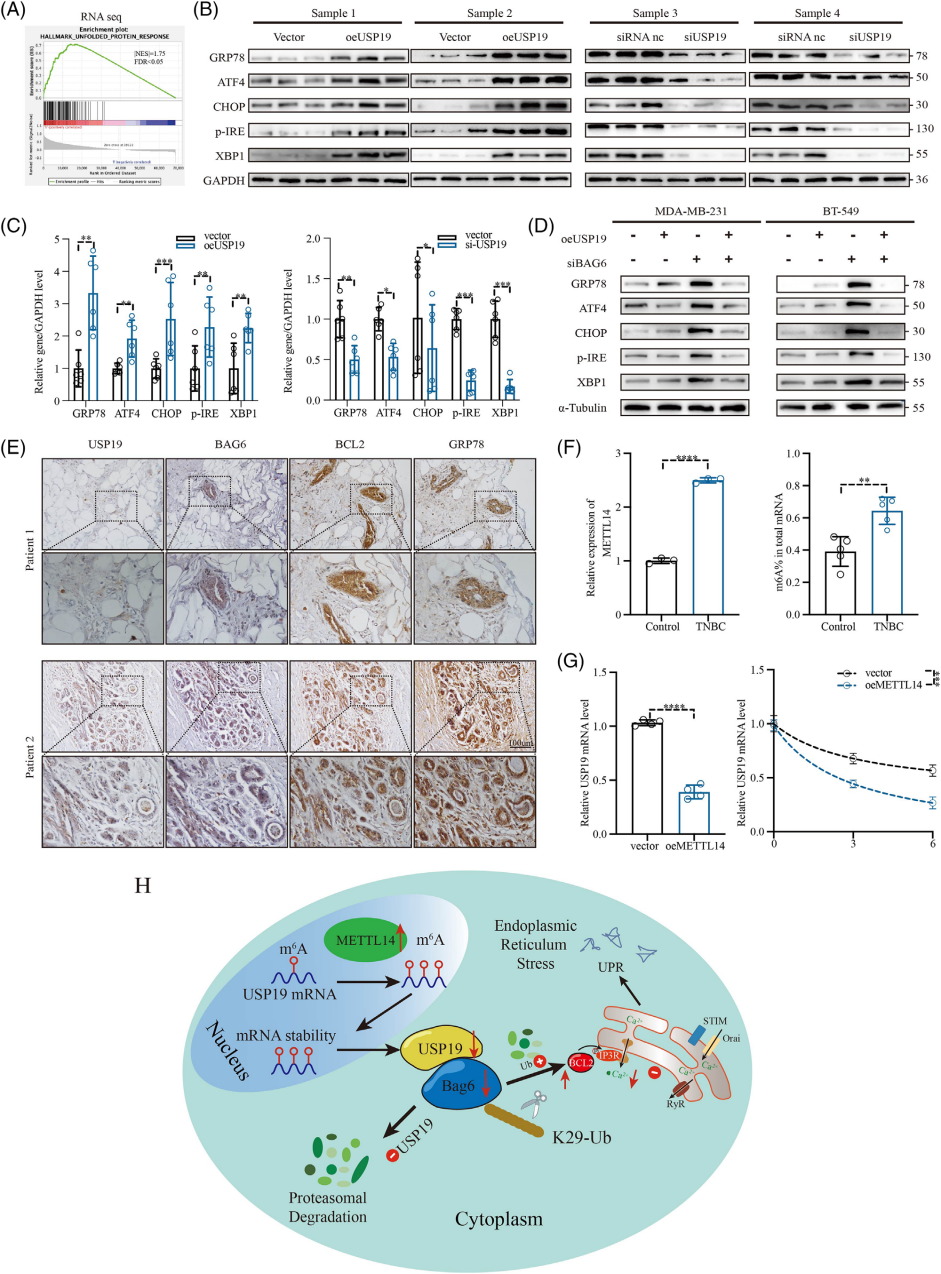
*USP19* induced BC apoptosis via endoplasmic reticulum stress (ERS). (A) Based on the sequencing data of 196 cases, gene set enrichment analysis (GSEA) revealed that UNFOLDED PROTEIN RESPONSE was enriched in the *USP19* high‐expression subgroup. (B,C) WB analysis of GRP78, ATF4, CHOP, p‐IRE and XBP1 in mouse tissue with *USP19* overexpression and knockdown. (D) WB analysis of GRP78, ATF4, CHOP, p‐IRE and XBP1 in MDA‐MB‐231 and BT‐549 with *USP19* overexpression and *BAG6* knockdown. (E) Representative images of IHC staining of USP19, BAG6, BCL2 and GRP78 in human BC specimens. The TUNEL staining in tumour sections. (F) Relative METTL14 levels in different tissues (Basal‐like and normal tissues); m^6^A levels in mRNAs of TNBC and paracancerous tissue. (G) Relative *USP19* mRNA levels in MDA‐MB‐231 transfected with vector or oe*METTL14*; RNA lifetime of *USP19* in indicated MDA‐MB‐231 cells as determined by monitoring transcript abundance after transcription inhibition (actinomycin D, 5 μg/mL). H. Working model for regulation of *BAG6* stability and endoplasmic reticulum stress by *USP19*. A representative dataset is displayed as mean ± SEM values. ns., not significant, *p < .05, **p < .01, ***p < .001.

According to gene set enrichment analysis, high expression of *USP19* appeared to be more correlated with the UPR (Figure 7A). *BAG6*, an abundant cytoplasmic chaperone, is related to the biogenesis of ER tail‐anchored membrane proteins, to the quality control of ribosome‐deficient plastids and mislocalised proteins, and acts to increase the efficiency of the ER‐associated protein degradation (ERAD) system. We investigated whether *USP19* influences TNBC development via *BAG6*‐mediated regulation of ER stress. To further investigate the effect of *USP19* on *BAG6*‐induced ERAD, we validated the expression levels of ATF4, CHOP, p‐IRE, GRP78 and XBP1 in animal models by western blotting (Figure 7B–7D). Histological and immunohistochemical (IHC) analyses found that *USP19* enhanced the expression of ER stress‐related proteins to induce the ER stress response (Figure 7E). Taken together, these results indicated that *USP19* can induce the activation of ER stress‐related apoptosis through stabilization of *BAG6* expression levels, thereby ameliorating TNBC progression in vivo.

### 3.8 *METTL14* reduced m^6^A RNA methylation of *USP19*

Lastly, after exploring the function of *USP19* in BC cells, we wondered why the expression of *USP19* was decreased in TBNC. *N*_6_‐methyladenosine (m^6^A) modification is the most classic intracellular mRNA post‐transcriptional modification and is involved in the regulation of various biological processes. We speculated that the m^6^A modification might be responsible for the downregulation of *USP19*. To test this hypothesis, we examined *METTL14* mRNA levels in TNBC tissues by qRT‐PCR, and discovered that the expression of *METTL14* was significantly upregulated in TNBC tissues (Figure 7F). A significant increase in the m^6^A level in mRNAs was also found in TNBC tissues (Figure 7F). Moreover, the *USP19* mRNA level was significantly down‐regulated after overexpression of *METTL14* (Figure 7G), and overexpression of *METTL14* markedly decreased the half‐life of *USP19* mRNA in MDA‐MB‐231 cells (Figure 7G). We treated MDA‐MB‐231 cells with cycloleucine, which has been extensively utilised in RNA methylation studies and might lower mRNA methylation. Cycloleucine was able to decrease the expression of USP19 without affecting METTL14 (Figure 5E,F). Collectively, the above results clarified that *METTL14*‐mediated m^6^A modification results for the downregulation of *USP19* mRNA levels. We treated MDA‐MB‐231 cells with cycloleucine, which has been extensively utilised in RNA methylation studies and might lower mRNA methylation. (Figure S3F).

## 4 DISCUSSION

Estrogen receptor, progesterone receptor (*PR*), and human epidermal growth factor receptor 2 (*HER2*) are lost in TNBC, which accounts for approximately 10%–20% of BCs.^34^ However, TNBC tended to be poorly differentiated and more prone to recurrence and metastasis than other BC subtypes. TNBC with recurrence and metastasis was more aggressive and less sensitive to radiotherapy and chemotherapy. Patients with advanced TNBC had the shortest median overall survival of approximately 18 months (13.6–20.1 months).^35^ Therefore, it was very important to clarify the pathogenesis and factors inducing TNBC. Using both in vitro and in vivo assays, we first showed that *USP19* was significantly downregulated in TNBC and that overexpression impaired colony formation ability in TNBC cell lines. *USP19* was shown to inhibit cell proliferation, while inducing apoptosis by reducing ubiquitination and degradation of BAG6 and enhancing BCL2 induced ER stress by elevating the intracellular Ca^2+^ concentration. Therefore, we identified *USP19* as a potential target for TNBC therapy and elucidated the possible mechanism by which *USP19* inhibits TNBC progression.

Ubiquitination is a reversible process in which deubiquitinases separate ubiquitin molecules from proteins to increase their stability.^36^, ^37^, ^38^ It has been reported to be critical for the regulation of programmed cell death.^39^, ^40^ A recent publication showed that *HOIL1* E3 mediates mono‐ubiquitination of the linear ubiquitin chain assembly complex, resulting in embryonic death upon knockout of *HOIL1* in mice. This process can be achieved by knockout of caspase‐8 and rescued by MLKL, suggesting the distinct interaction between cell death and ubiquitination.^41^ A study found that *PELI1* can promote K48‐linked ubiquitination of *RIP3* on Lys363, leading to proteasomal degradation, thereby inhibiting necroptosis.^42^ It was also demonstrated that *CYLD* promotes necroptosis after deubiquitination of *RIP1* in necrosomes.^38^, ^43^ USP37 can regulate BLM RecQ like helicase deubiquitination and stabilises its expression, thereby maintaining the DNA damage response in BC cells.^44^ Sun found that USP36 deubiquitinates c‐Myc in the nucleolus and acts downstream of c‐Myc to promote BC proliferation.^45^ *AKT* was found to promote nuclear export after phosphorylation of *USP4* and removes *TβRI* ubiquitination, promoting *TGFβ*‐induced epithelial‐to‐mesenchymal transition in BC.^46^ Deubiquitination thus plays a crucial role in the development of BC. In this study, we found, for the first time, that USP19 reduced ubiquitination and degradation of BAG6 to induce BC cell apoptosis and ER stress. *USP19* was reported to interact with *TRIF* and remove the K27‐linked polyubiquitin moiety of TRIF, thereby inhibiting *TRIF* recruitment to *TLR3/4* and promoting innate immune responses.^47^ Nevertheless, the functional role of *USP19* in cell biology, particularly in the regulation of ER stress, was still unclear.

It is reported that seven lysine residues (K6, K11, K27, K29, K33, K48, and K63) can be attached to the Homotypic Ub polymer's C‐terminus.^48^ Usually, K48‐linked ubiquitin chains are responsible for target proteins’ degradation. However, other ubiquitin chains also play important roles in the cellular function. Deubiquitinase (DUB) TRABID was shown to preferentially hydrolyse K29 and K33 links.^49^ Yu et al. revealed that UBE3C and TRABID, two ubiquitin ligases, cooperate reciprocally to regulate VPS34 through K29/K48‐branched ubiquitin chains.^50^ TRIP12 selectively assembles K29‐linked ubiquitin chains, boosting the development of K29/K48 branched ubiquitination.^51^ In this paper, we found that USP19 removed the K29‐linked ubiquitin chains of BAG6 and stabilised the latter's protein level.

ER stress is a highly dynamic process that may lead to abnormal apoptosis and death. Genetic alterations can also promote ER stress and persistent activation of the UPR in tumour cells. Excessive activation of tumour proto‐oncogenes could lead to increased protein synthesis, while proliferating cells require rapid ER amplification to divide into daughter cells.^52^ In addition, multiple factors, such as chemotherapy, induce a lethal ER stress response with immunogenic cell death triggers, which activates a protective antitumor immune response.^15^ Here, we showed that USP19 binds to BAG6 and decreases BAG6 ubiquitination, which prevents BAG6 from being degraded in TNBC tumour tissue and BC cells. Knockdown of *BAG6* in mice also reversed ER stress triggered by *USP19*, indicating that the essential effects of *USP19* on ER stress depend on BAG6 stability. Kawahara et al.^30^, ^53^ identified *BAG6* as a cytosolic interactor of misfolded proteins from ER by MS analysis, showing that *BAG6* specifically and abundantly bound to the deglycosylated version of the misfolded protein. Misfolded protein is known to be a key indicator of ER stress. These findings suggested that *USP19* controls ER stress, most likely through controlling BAG6 stability. Although the majority of our recent work has been focused on TNBC cell lines, *USP19* overexpression mice have allowed us to more thoroughly study and confirm the specific functional role of *USP19*. Therefore, we next investigated the molecular mechanism by which *USP19* induces changes in the ER stress by affecting the expression of *BAG6*.

*BCL2* is a prominent target of cutting‐edge treatment strategies for cancer and other disorders and was initially discovered through genetic research of B cell lymphomas.^54^, ^55^, ^56^ It is one of the most significant controllers of apoptosis. The *BCL2* family is often classified into two groups: pro‐survival BCL2 proteins (such as BCL2, A1, Bcl‐xL and Mcl1) and proapoptotic BCL2 proteins (such as Bad, Bax/Bak, Bim, Bik and Puma).^57^, ^58^ Signals coming from or focusing on intracellular organelles, such as the ER or the mitochondria, are frequently responsible for controlling the apoptosis process.^59^ Growing data suggest that the *BCL2* family regulates apoptosis in the ER, the major Ca^2+^ cellular storage organelle. BAG6 is a protein that resembles ubiquitin, according to Kawahara. In this paper, we showed that BAG6 binds to BCL2 and increases BCL2 ubiquitination, which decrease BCL2 accumulation in BC cells. Cytotoxic stress and cell death may be brought on by cellular Ca^2+^ excess or disruptions of intracellular Ca^2+^ storage.^33^ *IP3R* is a calcium channel that resides in the ER. Each subunit of the tetrameric IP3R protein comprises a transmembrane pore region at C‐terminus, a ligand binding domain at N‐terminus and an intervening modulatory domain.^60^ Numerous cellular activities, including metabolism, gene expression and apoptosis, are regulated by calcium release via the *IP3R*.^61^, ^62^ *BCL2* overexpression has been demonstrated to lower ER Ca^2+^ storage and inhibit capacitative Ca^2+^ entry following ER Ca^2+^ release.^63^, ^64^ It was found that BCL2's BH4 domain facilitated a connection with intracellular Ca^2+^ channel the inositol 1,4,5‐trisphosphate receptor (*IP3R*) to control cell growth and survival.^33^, ^60^, ^65^, ^66^ In MDA‐MB‐231 cells, endogenous *p‐IP3R* protein levels were decreased when *BCL2* was knocked down, while *IP3R* expression remained mostly unchanged. We discovered that ubiquitin‐like protein BAG6 can elevate the intracellular Ca^2+^ concentration to alter ER stress via regulating the level of BCL2 ubiquitination and decreasing IP3R phosphorylation modification level. Our findings implied that *USP19* can be a possible therapeutic target in BC, together with evidence revealing that decreased expression of *USP19* indicated a poorer prognosis in this disease.

Multiple perturbations to cells can lead to accumulation of unfolded proteins in the ER, which triggers the UPR when UPs accumulate to a certain amount.^67^, ^68^ When the primary stimuli that cause protein unfolding in the ER are prolonged or excessive, the adaptive mechanisms initiated by the UPR fail to compensate, and cell death by apoptosis will be triggered.^69^ Activated *IRE1α* promotes the expression of *XBP1* and other canonical UPR genes, such as foldase, chaperone, and ERAD machinery. *IRE1α*‐*XBP1* could regulate the degradation of misfolded proteins and enhance the folding and secretion of proteins to play an important role in the occurrence of the UPR. Through regulating the expression of key proteins, including lactate dehydrogenase A and glucose transporter 1, XBP1 binds to hypoxia‐inducible factor 1α (HIF1A) to regulate the hypoxia response and glycolysis in TNBC.^70^, ^71^, ^72^ Our results revealed that the levels of *GRP78, ATF4, CHOP, p‐IRE*, and *XBP1* expression were increased in mouse tumour tissue overexpressing *USP19*, indicating an increased ER stress parallel to the expression of *USP19* in TNBC tumorigenesis. ER stress‐related proteins were also significantly up‐regulated in human BC cells overexpressing *USP19* compared with normal controls. Collectively, *USP19* can significantly increase the unfolded protein entering the ER and induce ER stress, by reducing BAG6 ubiquitination to kill TNBC cells.

m^6^A methylation had received a lot of attention recently and was now widely acknowledged as the most frequent and prolific alteration on mRNA and as a mechanism to control RNA translation, stability, and degradation.^73^, ^74^ We found that total m^6^A levels were upregulated in TNBC tissues, along with elevated m^6^A “writer” METTL14 levels. Sun et al.^75^ reported that *LNC942* drew METTL14 protein directly due to its unique recognition sequence (+176 to +265). By modifying post‐transcriptional m^6^A, this stabilises the downstream expression of *LNC942*, including *CXCR4* and *CYP1B1*. To determine whether m^6^A modification was associated with the regulation of *USP19* mRNA, we overexpressed *METTL14* in MDA‐MB‐231, and found that the expression of *USP19* mRNA was decreased when *METTL14* was overexpressed. RNA stability experiments demonstrated that the stability of *USP19* mRNA was correspondingly decreased when *METTL14* was overexpressed. In an initial investigation of the upstream regulatory mechanism of *USP19*, we discovered that m^6^A modification of *USP19* resulted in *USP19* downregulation. Further detailed investigations are needed in subsequent studies to fully confirm this conclusion.

In conclusion, this study showed that *USP19* played a key role in TNBC cancer proliferation and apoptosis. The pace of mouse tumour development was considerably reduced by targeting U*SP19*. Additionally, our research identified a novel mechanism by which *USP19* elevates intracellular Ca^2+^ levels to induce ER stress in TNBC, through regulating the ubiquitination and degradation of BAG6 and BCL2. Additionally, we demonstrated that *USP19* was downregulated at the mRNA level, as a result of increased m^6^A alteration. The bioinformatics analysis and our patient data, in particular, improved the relevance and dependability of the findings on *USP19* and its associated pathways. Since only deubiquitinases were examined in this investigation, it was possible that additional functional proteins may play a role in the control of ER stress and apoptosis in TNBC mice. Our findings indicated that targeting USP19 /BAG6/BCL2 axis may be an efficient therapeutic target for treating TNBC.

## CONFLICT OF INTEREST STATEMENT

The authors declare that they have no known competing financial interests or personal relationships that could have appeared to influence the work reported in this paper.

## Supporting information


**Figure S1** *USP19* and *USP19* (C607S) affect cell proliferation and cell apoptosis. (A) Effects of *USP19* knockdown on the colony formation of BC cells. (B) Representative profiles of EdU cell growth in MDA‐MB‐231 cells and BT‐549 cells after transfection with si*USP19* respectively compared with the control. (C) CCK‐8 was used to determine the proliferation of BC cells transfected with si*USP19*. OD value between si*USP19* plasmid and corresponding control group was significantly different. The data expressed as the mean ± SD. (D) Evaluation of the impact of altered *USP19* expression on cell apoptosis. (E) CCK‐8 was used to determine the proliferation of BC cells transfected with Myc‐*USP19^C607S^* plasmid. OD value between Myc‐*USP19^C607S^* plasmid and corresponding control group was no different. (F) qRT‐PCR and Western blot was used to verify the expression of *USP19* in cells transfected with Myc‐*USP19* plasmid and *USP19* siRNA, respectively. The data expressed as the mean ± SD. Statistical results are quantified by ImageJ software. A representative dataset is displayed as mean ± SEM values. ns, Not significant, *p < .05, **p < .01, ***p < .001.

**Figure 2** (A) qRT‐PCR was used to verify the evidence that we stably transfected cell lines for in vivo experiments. (B) *USP19* mRNA is unable to bind *BAG6* protein according to RNA pulldown and immunoprecipitation assays. (C) Kaplan–Meier curves showed the stratification analysis of the *USP19* in BC tissues from TCGA. (D) Exogenous protein interactions were confirmed in HEK‐293T cells. Lysates from HEK‐293T cells transfected with Myc‐tagged *USP19* and Flag‐tagged *BAG6* plasmids were immunoprecipitated with anti‐Flag or anti‐Myc, respectively, and assessed by immunoblotting with anti‐Myc (*USP19*) and anti‐Flag (*BAG6*). (E) *BAG6* protein levels in control siRNA and *USP19* siRNA HEK‐293T were evaluated by immunoblotting with anti‐BAG6 and anti‐USP19 in the presence of cycloheximide (CHX, 10 μg/mL) for indicated timepoint. A representative dataset is displayed as mean ± SEM values. ns., Not significant, *p < .05, **p < .01, ***p < .001.

**FIGURE 3** (A) The effect of USP19 up‐regulation in the *BAG6* mRNA level. (B) Analysis of BAG6 and USP19 protein levels by Western blot in MDA‐MB‐231 transfected with si*USP19* with lysosome inhibitor Chloroquine (CQ). (C) CCK‐8 was used to determine the proliferation of BC cells transfected with *BAG6* plasmid. OD value has no significant difference between *BAG6* plasmid and corresponding control group. (D) Photographs of tumours obtained from the different groups of nude mice transfected with BAG6 plasmid, respectively. (E) Fluorescence microscopy was used to observe Fluo‐4 AM‐loaded MDA‐MB‐231 and BT549 cells. Fluo‐4 fluorescence (green) increases with intracellular Ca^2+^ concentration. The data expressed as the mean ± SD. F. The results of qRT–PCR showing the expression of *METTL14* mRNA and *USP19* mRNA in MDA‐MB‐231 cells. The low concentration of cycloleucine was 10 mM, the middle was 20 mM, and the high was 40 mM. A representative dataset is displayed as mean ± SEM values. ns., Not significant, *p < .05, **p < .01, ***p < .001.

Supporting Information

Supporting Information

Supporting Information

## Data Availability

The datasets used and/or analysed during the current study are available from the corresponding author on reasonable request. Supporting information accompanies this paper at Supplementary Tables and Materials.
