# Supplementary figures and images for: Deubiquitinase USP19 modulates apoptotic calcium release and endoplasmic reticulum stress by deubiquitinating BAG6 in triple negative breast cancer
Source: Clin Transl Med. 2023 Sep 12;13(9):e1398. doi: 10.1002/ctm2.1398 (PMC10497826; doi:10.1002/ctm2.1398)

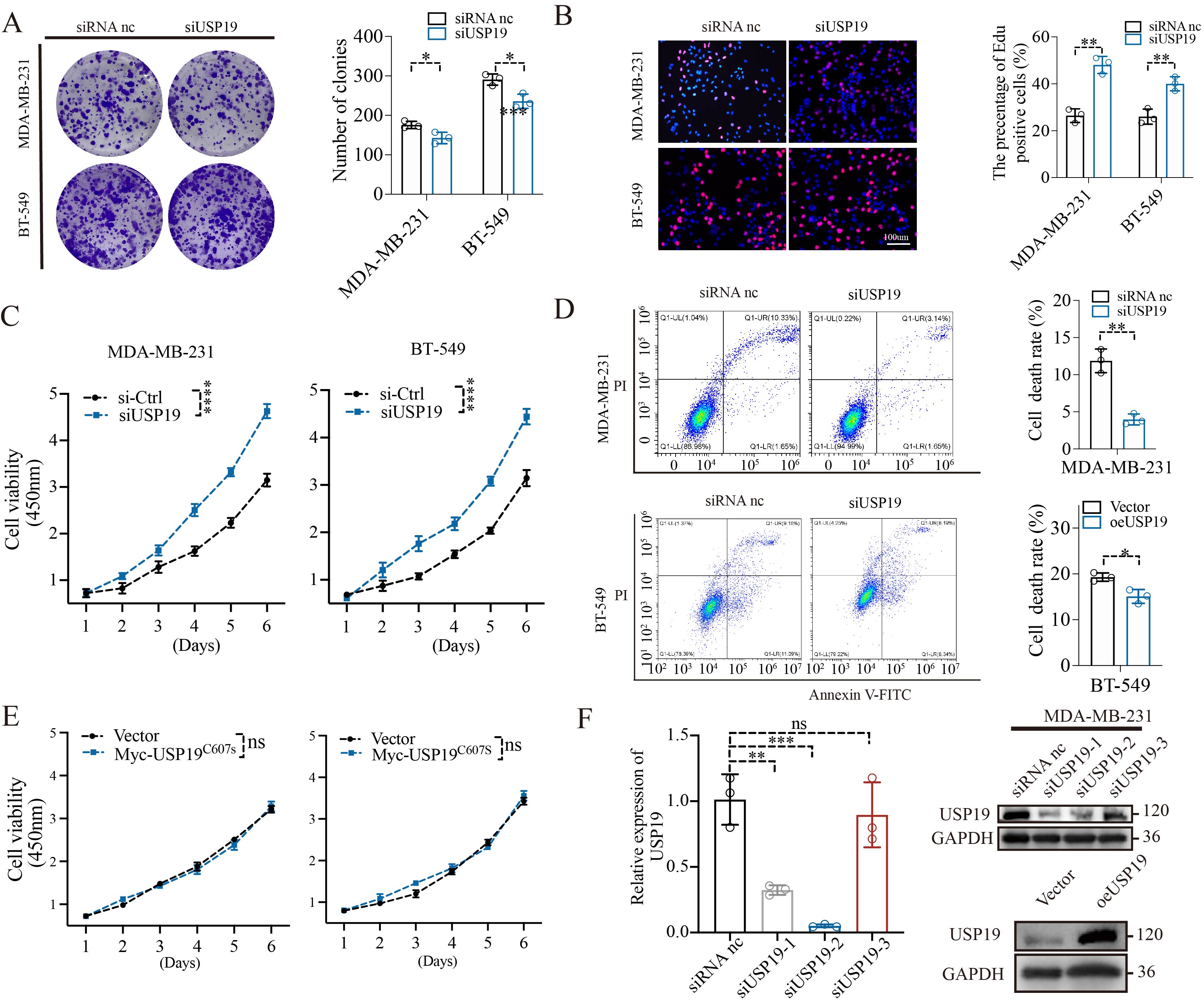

Supplement: Supplementary file 1 — Figure S1 USP19 and USP19 (C607S) affect cell proliferation and cell apoptosis. (A) Effects of USP19 knockdown on the colony formation of BC cells. (B) Representative profiles of EdU cell growth in MDA‐MB‐231 cells and BT‐549 cells after transfection with siUSP19 respectively compared with the control. (C) CCK‐8 was used to determine the proliferation of BC cells transfected with siUSP19. OD value between siUSP19 plasmid and corresponding control group was significantly different. The data expressed as the mean ± SD. (D) Evaluation of the impact of altered USP19 expression on cell apoptosis. (E) CCK‐8 was used to determine the proliferation of BC cells transfected with Myc‐USP19C607S plasmid. OD value between Myc‐USP19C607S plasmid and corresponding control group was no different. (F) qRT‐PCR and Western blot was used to verify the expression of USP19 in cells transfected with Myc‐USP19 plasmid and USP19 siRNA, respectively. The data expressed as the mean ± SD. Statistical results are quantified by ImageJ software. A representative dataset is displayed as mean ± SEM values. ns, Not significant, *p < .05, **p < .01, ***p < .001. [file CTM2-13-e1398-s001.jpg]

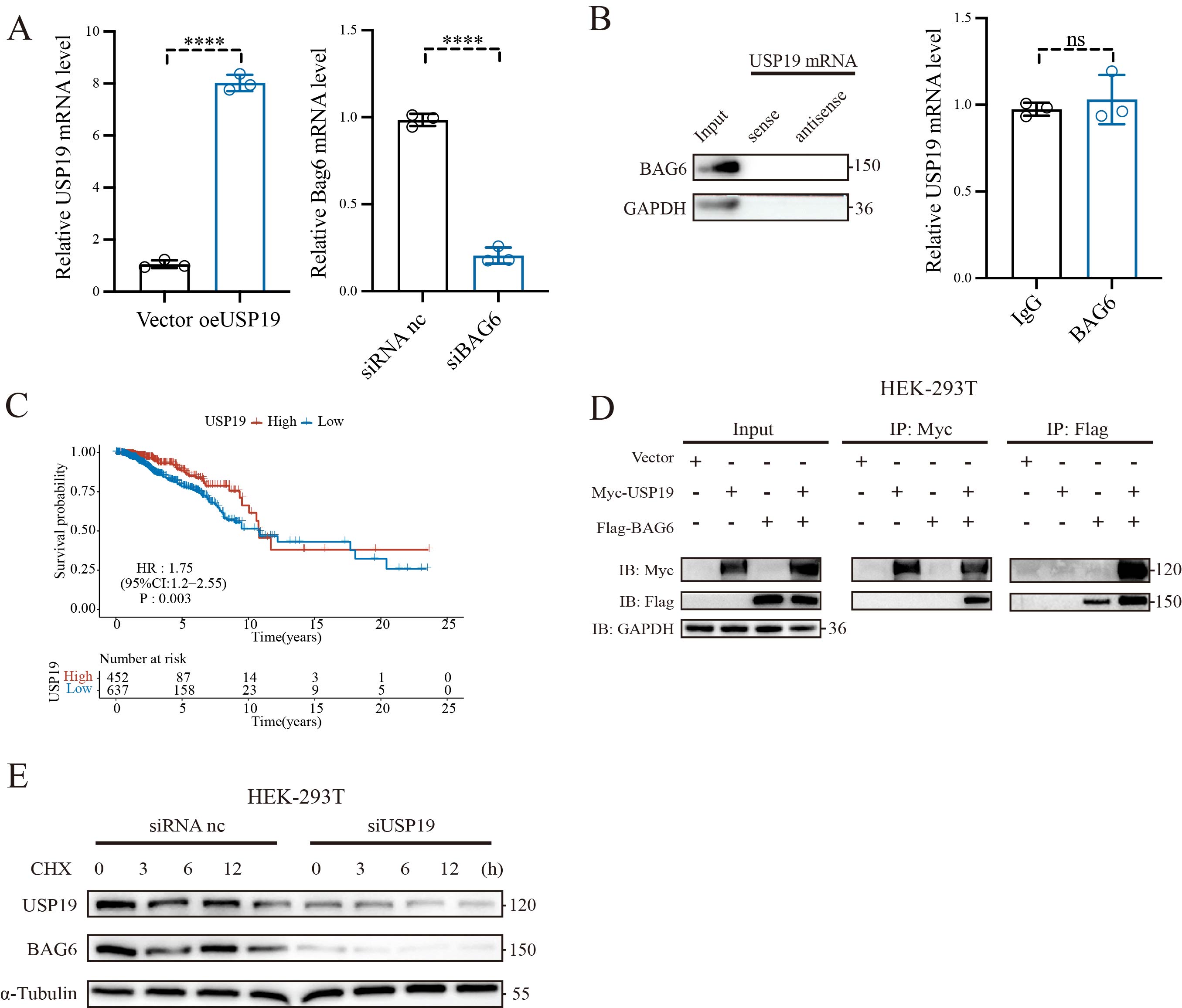

Supplement: Supplementary file 2 — Figure 2 (A) qRT‐PCR was used to verify the evidence that we stably transfected cell lines for in vivo experiments. (B) USP19 mRNA is unable to bind BAG6 protein according to RNA pulldown and immunoprecipitation assays. (C) Kaplan–Meier curves showed the stratification analysis of the USP19 in BC tissues from TCGA. (D) Exogenous protein interactions were confirmed in HEK‐293T cells. Lysates from HEK‐293T cells transfected with Myc‐tagged USP19 and Flag‐tagged BAG6 plasmids were immunoprecipitated with anti‐Flag or anti‐Myc, respectively, and assessed by immunoblotting with anti‐Myc (USP19) and anti‐Flag (BAG6). (E) BAG6 protein levels in control siRNA and USP19 siRNA HEK‐293T were evaluated by immunoblotting with anti‐BAG6 and anti‐USP19 in the presence of cycloheximide (CHX, 10 μg/mL) for indicated timepoint. A representative dataset is displayed as mean ± SEM values. ns., Not significant, *p < .05, **p < .01, ***p < .001. [file CTM2-13-e1398-s006.jpg]

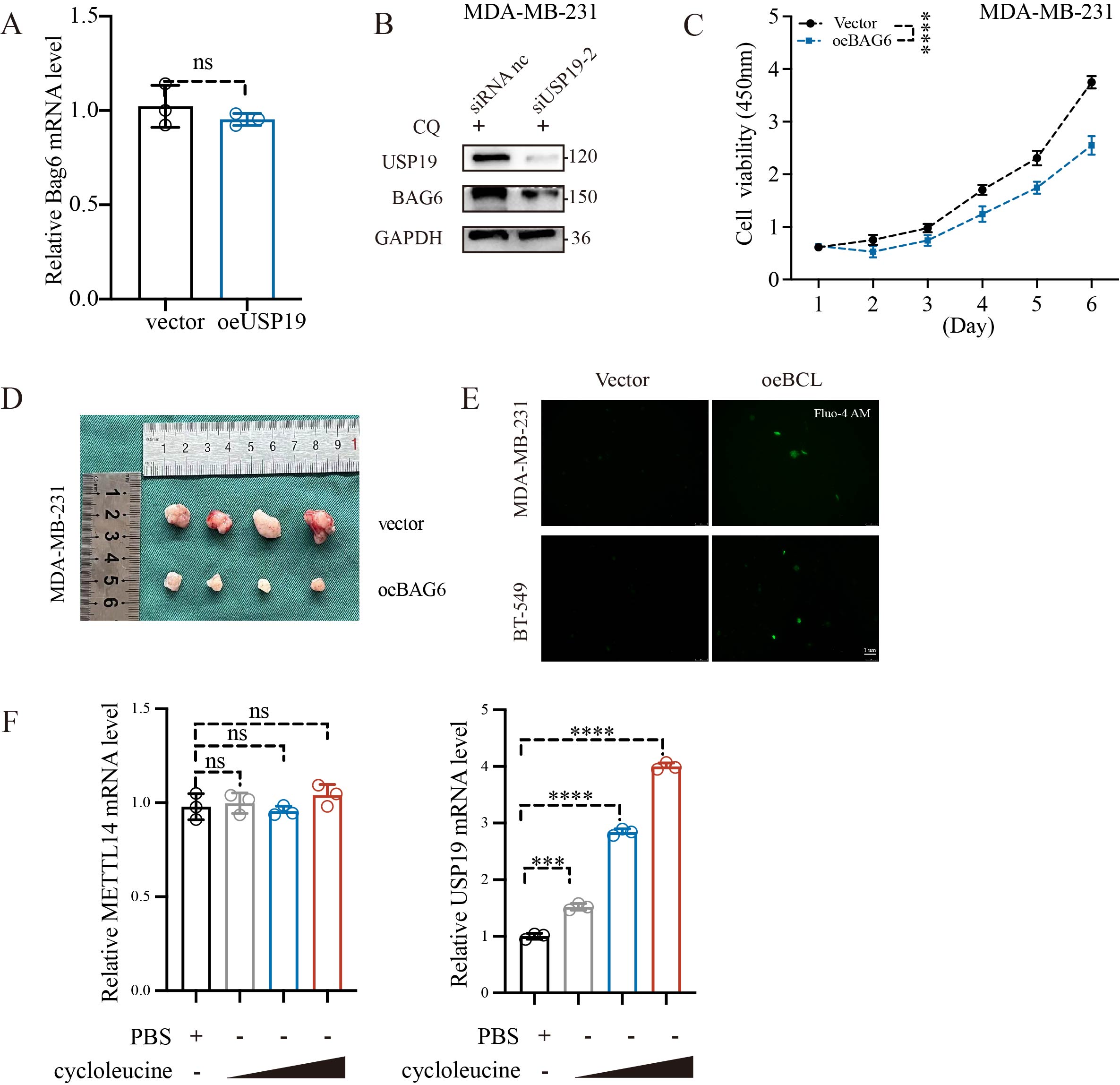

Supplement: Supplementary file 3 — FIGURE 3 (A) The effect of USP19 up‐regulation in the BAG6 mRNA level. (B) Analysis of BAG6 and USP19 protein levels by Western blot in MDA‐MB‐231 transfected with siUSP19 with lysosome inhibitor Chloroquine (CQ). (C) CCK‐8 was used to determine the proliferation of BC cells transfected with BAG6 plasmid. OD value has no significant difference between BAG6 plasmid and corresponding control group. (D) Photographs of tumours obtained from the different groups of nude mice transfected with BAG6 plasmid, respectively. (E) Fluorescence microscopy was used to observe Fluo‐4 AM‐loaded MDA‐MB‐231 and BT549 cells. Fluo‐4 fluorescence (green) increases with intracellular Ca2+ concentration. The data expressed as the mean ± SD. F. The results of qRT–PCR showing the expression of METTL14 mRNA and USP19 mRNA in MDA‐MB‐231 cells. The low concentration of cycloleucine was 10 mM, the middle was 20 mM, and the high was 40 mM. A representative dataset is displayed as mean ± SEM values. ns., Not significant, *p < .05, **p < .01, ***p < .001. [file CTM2-13-e1398-s004.jpg]
