# Supplementary material for: Deubiquitinase USP19 modulates apoptotic calcium release and endoplasmic reticulum stress by deubiquitinating BAG6 in triple negative breast cancer
Source: Clin Transl Med. 2023 Sep 12;13(9):e1398. doi: 10.1002/ctm2.1398 (PMC10497826; doi:10.1002/ctm2.1398)
Supplement: Supplementary file 4 — Supporting Information [file CTM2-13-e1398-s002.docx]

**Supplementary Table. Primers and Plasmid used for Quantitative RT-PCR or transfection**

| RNA | 5'to 3' |
| --- | --- |
| Myc-USP19 | miaolingbio, China (Cat# P24609) |
| Flag-BAG6 | Public Protein/Plasmid Library (Cat#7917) |
| Myc-USP19^C607S^ | NA |
| USP19 | F: ATGGTGAACCTGGCGTTTGTC |
|  | R: GGTCTGGAAGATGAGCGTGAAGT |
| siUSP19-1 | F: caccgCAGGTTTGGGAGTTCAGCGG  R: aaacCCGCTGAACTCCCAAACCTGc |
| siUSP19-2 | F: caccgAGGGTTTCTGCTCCGGACCA  R: aaacTGGTCCGGAGCAGAAACCCTc |
| siUSP19-3 | F: caccGAAGGGCCCGGATTCAGTGG  R: aaacCCACTGAATCCGGGCCCTTC |
| siRNA nc | F: UUCUUCGAACGUGUCACGUTT  R: ACGUGACACGUUCGGAGAATT |
| BAG6 | F: TCAGCTGAGACAGAACCTTGG  R: TCCCCTGATGAGGAAGGGCCA |
| siBAG6 | F: TTTCTCCAAGAGCAGTTTA  R: ATGATGCACATGAACATTC |
| siBcl-2 | CGGGAGATAGTGATGAAGTACATCCATTA |
| HA-K6-Ubiquitin | miaolingbio, China (Cat# P31806) |
| HA-K11-Ubiquitin | miaolingbio, China (Cat# P31913) |
| HA-K27-Ubiquitin | miaolingbio, China (Cat# P31987) |
| HA-K29-Ubiquitin | miaolingbio, China (Cat# P31814) |
| HA-K33-Ubiquitin | miaolingbio, China (Cat# P31798) |
| HA-K48-Ubiquitin | miaolingbio, China (Cat# P31802) |
| HA-K63-Ubiquitin | miaolingbio, China (Cat# P31800) |
| METTL14 | F: GAACACAGAGCTTAAATCCCCA  R: TGTCAGCTAAACCTACATCCCTG |
| oeMETTL14 | miaolingbio, China (Cat# P22487) |

**Supplementary Table. Antibodies for Western blot, immunoprecipitation and immunofluorescence.**

| Antibodies |  |
| --- | --- |
| USP19 | Proteintech Group (Cat#25768-1-AP) |
| BAG6 | Proteintech Group (Cat#26417-1-AP) |
| IgG | Proteintech Group (Cat# 30000-0-AP) |
| Flag-tag | SIGMA (Cat# F1804) |
| Myc-tag | Proteintech Group (Cat# 16286-1-AP) |
| GAPDH | Proteintech Group (Cat# 60004-1-Ig) |
| α-Tubulin | Proteintech Group (Cat# 11224-1-AP) |
| HA-tag | Proteintech Group (Cat# 66006-2-Ig) |
| GRP78 | Proteintech Group (Cat# 11587-1-AP) |
| ATF4 | Proteintech Group (Cat# 10835-1-AP) |
| CHOP | Proteintech Group (Cat# 15204-1-AP) |
| phosphor-(p-)IRE | Proteintech Group (Cat# 27528-1-AP) |
| XBP1 | Proteintech Group (Cat# 24868-1-AP) |
| Bcl-2 | Proteintech Group (Cat# 26593-1-AP) |
| IP3R | Cell Signaling Technology (Cat# 8568) |
| p-IP3R | Cell Signaling Technology (Cat# 8548) |
